# Coinhibition of topoisomerase 1 and BRD4-mediated pause release selectively kills pancreatic cancer via readthrough transcription

**DOI:** 10.1126/sciadv.adg5109

**Published:** 2023-10-13

**Authors:** Donald P. Cameron, Jan Grosser, Swetlana Ladigan, Vladislav Kuzin, Evanthia Iliopoulou, Anika Wiegard, Hajar Benredjem, Kathryn Jackson, Sven T. Liffers, Smiths Lueong, Phyllis F. Cheung, Deepak Vangala, Michael Pohl, Richard Viebahn, Christian Teschendorf, Heiner Wolters, Selami Usta, Keyi Geng, Claudia Kutter, Marie Arsenian-Henriksson, Jens T. Siveke, Andrea Tannapfel, Wolff Schmiegel, Stephan A. Hahn, Laura Baranello

**Affiliations:** ^1^ Department of Cell and Molecular Biology, Karolinska Institutet, Stockholm, Sweden.; ^2^ Ruhr University Bochum, Faculty of Medicine, Department of Molecular GI Oncology, Bochum, Germany.; ^3^ Ruhr University Bochum, Knappschaftskrankenhaus, Department of Internal Medicine, Bochum, Germany.; ^4^ Division of Solid Tumor Translational Oncology, German Cancer Consortium (DKTK, partner site Essen) and German Cancer Research Center, DKFZ, Heidelberg, Germany.; ^5^ Bridge Institute of Experimental Tumor Therapy, West German Cancer Center, University Hospital Essen, Essen, Germany.; ^6^ Ruhr University Bochum, Knappschaftskrankenhaus, Department of Surgery, Bochum, Germany.; ^7^ Department of Internal Medicine, St. Josef-Hospital, Dortmund, Germany.; ^8^ Department of Visceral and General Surgery, St. Josef-Hospital, Dortmund, Germany.; ^9^ Department of Microbiology, Tumor and Cell Biology, Karolinska Institutet, Stockholm, Sweden.; ^10^ Science for Life Laboratory, Karolinska Institutet, Stockholm, Sweden.; ^11^ Institute of Pathology, Ruhr University Bochum, Bochum, Germany.

## Abstract

Pancreatic carcinoma lacks effective therapeutic strategies resulting in poor prognosis. Transcriptional dysregulation due to alterations in KRAS and MYC affects initiation, development, and survival of this tumor type. Using patient-derived xenografts of KRAS- and MYC-driven pancreatic carcinoma, we show that coinhibition of topoisomerase 1 (TOP1) and bromodomain-containing protein 4 (BRD4) synergistically induces tumor regression by targeting promoter pause release. Comparing the nascent transcriptome with the recruitment of elongation and termination factors, we found that coinhibition of TOP1 and BRD4 disrupts recruitment of transcription termination factors. Thus, RNA polymerases transcribe downstream of genes for hundreds of kilobases leading to readthrough transcription. This occurs during replication, perturbing replisome progression and inducing DNA damage. The synergistic effect of TOP1 + BRD4 inhibition is specific to cancer cells leaving normal cells unaffected, highlighting the tumor’s vulnerability to transcriptional defects. This preclinical study provides a mechanistic understanding of the benefit of combining TOP1 and BRD4 inhibitors to treat pancreatic carcinomas addicted to oncogenic drivers of transcription and replication.

## Teaser

TOP1 and BRD4 drugs synergize to kill pancreatic cancer in vivo via readthrough transcription without emergent drug resistance.

## INTRODUCTION

Dysregulated transcriptional programs cause cancer cells to become highly dependent on certain regulators of gene expression (*1*–*5*). This dependency may provide opportunities for promising therapeutic interventions. Pancreatic ductal adenocarcinoma (PDAC) is a highly lethal malignancy due to the lack of early diagnosis and limited response to treatments, with patients with PDAC having a 5-year survival of 9% (*6*). Most PDACs harbor oncogenic KRAS mutations and elevated MYC signaling leading to dysregulation of global transcription and proliferation (*7*, *8*), potentially sensitizing cancer cells to therapeutic targeting with transcriptional inhibitors.

Following transcription initiation, the RNA polymerase II (RNAPII) pauses because of factors that (i) affect the stability of the elongation complex and the efficiency of nucleotide incorporation (*9*) and (ii) provide a physical obstacle for the movement of RNAPII (*10*, *11*). The stages preceding and following pausing are associated with modifications of the RNAPII C-terminal domain (CTD). This CTD “code” defines mRNA splicing, elongation, histone methylation, and polyadenylation via an array of dynamic interactions (*12*). The chromatin reader bromodomain-containing protein 4 (BRD4) facilitates pause release via two independent pathways: by recruiting the positive transcription elongation factor b (PTEF-b) complex on the RNAPII (*13*) and by enhancing the enzymatic activity of topoisomerase 1 (TOP1) via phosphorylation of RNAPII CTD on serine-2 (Ser-2) (*14*, *15*). TOP1 removes supercoiling in the DNA by transiently breaking one strand to allow controlled DNA rotation around the unbroken strand and resealing the break (*16*). According to the “twin domain model” (*17*), as DNA twists through the active site of an elongating RNAPII, positive supercoils are generated ahead, and negative supercoils trail behind the polymerase. Unless removed, this supercoiling will eventually halt the RNAPII. We have previously found a mechanism through which the RNAPII regulates TOP1 activity to favor transcription elongation. A positive feedback loop is established between RNAPII and TOP1 through their physical interaction. Upon BRD4-dependent phosphorylation (*15*), the RNAPII-CTD directly stimulates TOP1 beyond its intrinsic activity to remove the supercoiling that would otherwise oppose pause release (*14*). BRD4-stimulated RNAPII-CTD phosphorylation is also reported to be involved in the recruitment of transcription termination factors (TTFs) to facilitate timely termination (*18*). These TTFs, including cleavage and polyadenylation specificity factor (CPSF) and 64-kDa cleavage stimulation factor (CSTF64), are loaded onto RNAPII in a BRD4-dependent manner at the 3′ end of genes. CPSF and CSTF64 then promote the dephosphorylation of the elongation factor SPT5 by the protein phosphatase 1 nuclear targeting subunit complex, thus slowing the RNAPII and enabling DNA disengagement (*19*).

Although BRD4 belongs to the bromodomain and extraterminal domain (BET) family—a group of proteins known to interact with acetylated histones—the stimulation of TOP1 activity by BRD4 via RNAPII-CTD phosphorylation is independent of nucleosome acetylation (*14*). Thus, BRD4 acts through a bromodomain-dependent arm to drive PTEF-b on the pausing site and through a bromodomain-independent arm to stimulate TOP1. The simultaneous targeting of BRD4 and TOP1 with a panel of BET inhibitors and TOP1 poisons, respectively, synergistically inhibited cancer cell growth in vitro (*14*). The combined inhibition of both arms could be used therapeutically to target the transcriptional addiction of PDACs dependent on KRAS and MYC dysregulated gene expression programs (*2*).

Here, we tested the efficacy of this combinatorial strategy in vivo using a collection of patient-derived xenograft (PDX) mouse models for PDAC. We also included a pancreatic neuroendocrine carcinoma (panNEC) harboring a KRAS mutation and elevated MYCN, the MYC isoform expressed in neurons. PanNECs represent a poorly differentiated and highly malignant subgroup of neoplasms of the neuroendocrine pancreas. Although panNECs are rare malignancies (*20*), their incidence has increased steadily, especially as metastatic disease (*21*). The TOP1 poison irinotecan is already used to treat PDACs and panNECs as part of the FOLFIRINOX and FOLFIRI drug cocktails, respectively (*22*, *23*). However, while this treatment provided median overall survival of 11 months, it was associated with considerable side effects reducing patient quality of life. Since synergistic drug combinations enable reduced dosage while retaining efficacy, combining TOP1 and BRD4 inhibitors might represent a promising strategy to pharmacologically uncouple the TOP1-RNAPII regulation of transcription elongation, while reducing toxic nonspecific DNA damage associated with classical TOP1 inhibitors (*24*). We show that combining the TOP1 inhibitor irinotecan with the BRD4 inhibitor JQ1 synergistically kills the tumor cells in the PDX model of both tumor types. Mechanistically, we found that the inhibition of both bromodomain-dependent and -independent pathways profoundly impairs promoter-proximal pausing, transcriptional elongation, and the downstream mechanisms of termination, leading to readthrough transcription. Thus, transcribing RNAPIIs remain engaged with DNA for hundreds of kilobases into largely gene-free late replication regions, causing replication stress and inducing DNA damage and cellular stress signaling. Our results demonstrate that the synergistic drug combination selectively affects tumor viability based on their transcriptional addiction, leaving normal cells largely unaffected.

## RESULTS

### Combined inhibition of BRD4 and TOP1 is synergistic in killing xenografted tumors of pancreatic cancer

**Fig. 1.**
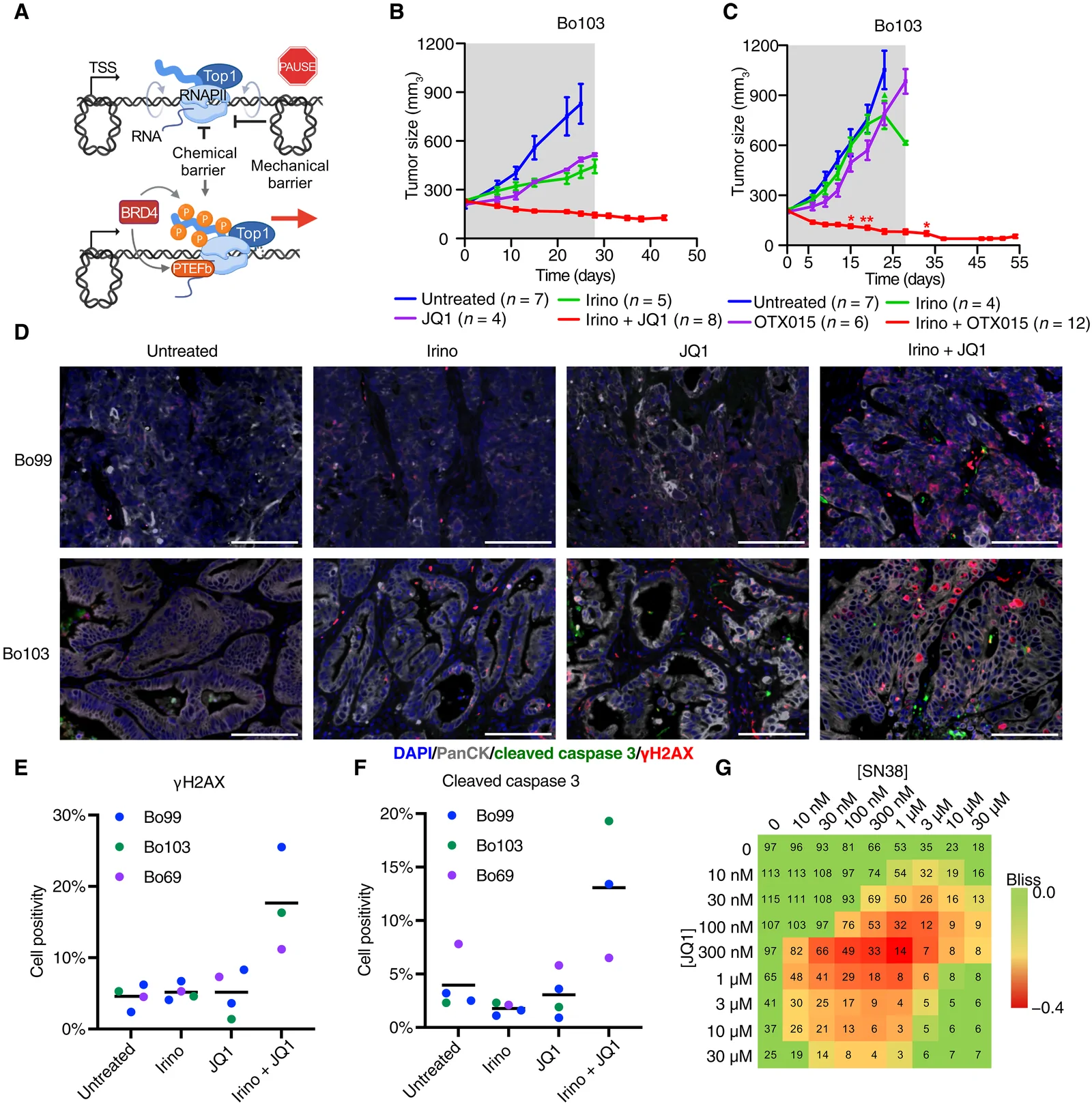
Combined drug treatment of topoisomerase 1 (TOP1) and bromodomain-containing protein 4 (BRD4) inhibitors shows synergy by killing pancreatic tumor cells both in vivo and in vitro. (**A**) Scheme describing TOP1 regulation during pause release based on (*14*). BRD4 and TOP1 activities are required to overcome promoter-proximal pausing and enable efficient transcription elongation. (**B**) Primary responses observed upon 28 days of treatment (shaded area) for the pancreatic ductal adenocarcinoma (PDAC) patient-derived xenograft (PDX) model Bo103 treated with irinotecan (Irino) and JQ1 by intraperitoneal injection, alone or in combination in comparison to untreated controls. Growth curves are derived from mean values ± SEM (error bars). (**C**) Same as (B), but with the bromodomain-containing protein 4 (BRD4) inhibitor OTX015. Each asterisk represents a mouse that was taken out of the treatment cohort at the indicated time point because of health issues of the animal. The triangle indicates a mouse taken out of the experiment because one of the two tumors reached the maximum size criteria. Two mice with altogether four tumors were kept for follow-up beyond end of treatment on day 28 to assess tumor recurrency. (**D**) Representative immunohistochemistry images of Bo99 and Bo103 PDX tumor sections treated for 5 days with irinotecan and/or JQ1 stained for DNA [4′,6-diamidino-2-phenylindole (DAPI), blue], PanCK (gray), cleaved caspase 3 (green), and γH2AX (red). Scale bars, 100 μm. (**E** and **F**) Quantitation of nuclear γH2AX (E) and cellular cleaved caspase 3 (F) positivity from samples in (D) and fig. S1D. (**G**) Checkerboard assay of cultured Bo103 cells treated with SN38 and JQ1 in combination as indicated. The percentage of confluency after treatment is denoted by the numbers in the squares. Synergy determined using the delta Bliss model of additivity with more negative values showing stronger synergy (visualized by red/green color coding). Representative checkerboard of *n* = 3.

We previously demonstrated that targeting two independent arms of promoter-proximal pausing regulation through inhibition of TOP1 and BRD4 (Fig. 1A) synergistically inhibited tumor growth in vitro (*14*). To determine whether this combination could effectively arrest cancer progression in vivo, we treated mice with implanted pancreatic cancer with the clinically approved TOP1 poison irinotecan (15 mg/kg, three times weekly, every second week), the BRD4 inhibitor JQ1 (50 mg/kg, daily), or both drugs in combination by intraperitoneal (i.p.) injection. Notably, while the JQ1 dose is in accordance with other studies, the irinotecan dose used in our study is considerably lower than common administration regimes of 40 to 300 mg/kg daily (*25*, *26*) to limit the nonspecific cytotoxic effects associated with TOP1 drugs.

Four PDX models with activated oncogenic KRAS mutations and elevated MYC isoforms (table S1), known to dysregulate global transcription (*7*), were chosen. Each monotherapy induced tumor growth reduction without provoking tumor regression (Fig. 1B and fig. S1, A to C). However, the combination therapy induced detectable tumor regression (mean maximal tumor volume reduction of on average 32 to 49% relative to the mean tumor volume at therapy start) after 28 days in the three PDAC PDX models, belonging either to the classical subtype (Bo103) and or to the quasi-mesenchymal subtype (Bo69 and Bo85) (*27*), the latter subtype being frequently linked to therapeutic resistance. Notably, the panNEC PDX model (Bo99) showed a near 100% tumor volume reduction. The synergism was also prominent when irinotecan was combined with OTX015, a JQ1 analog and clinical stage bromodomain inhibitor, indicating that this drug-targeting strategy could prove promising in patients with cancer (Fig. 1C). Immunohistochemistry staining of Bo69, Bo99, and Bo103 tumor sections demonstrated an increase in DNA damage response marker γH2AX and apoptotic marker cleaved caspase 3 after irinotecan + JQ1 treatment (Fig. 1, D to F, and fig. S1D). This effect was most pronounced in the Bo99 and Bo103 tumors, as the Bo69 section had high cleaved caspase 3 background signal. Given that the irinotecan treatment alone showed no increase in γH2AX relative to the untreated samples (Fig. 1E), the DNA damage signaling must be specific to the combination treatment, as opposed to the genotoxic properties of irinotecan. These in vivo experiments strongly indicate that the irinotecan + JQ1 treatment synergistically induces targeted DNA damage, apoptosis, and tumor shrinkage.

Of the tumors tested, Bo103 was the most adaptable for cell culture. We confirmed that the in vitro coinhibition of BRD4 and TOP1 with JQ1 and SN38, the active metabolite of irinotecan required for cell culture (*28*, *29*), was synergistic in killing cells as determined by the Bliss independence model of additivity (*30*), while no synergy was observed in the normal immortalized pancreatic cell line hTERT-HPNE (Fig. 1G and fig. S1E). Approximately 90% growth inhibition was detected after 48 hours of combined treatment with 500 nM SN38 and 1 μM JQ1, whereas individual administration had limited growth inhibitory effects (Fig. 1G and fig. S1F). Therefore, the Bo103 cells and these drug concentrations were subsequently used to characterize the tumor-specific mechanisms underlying the synergy.

### SN38 and JQ1 combination treatment synergistically inhibits transcription

**Fig. 2.**
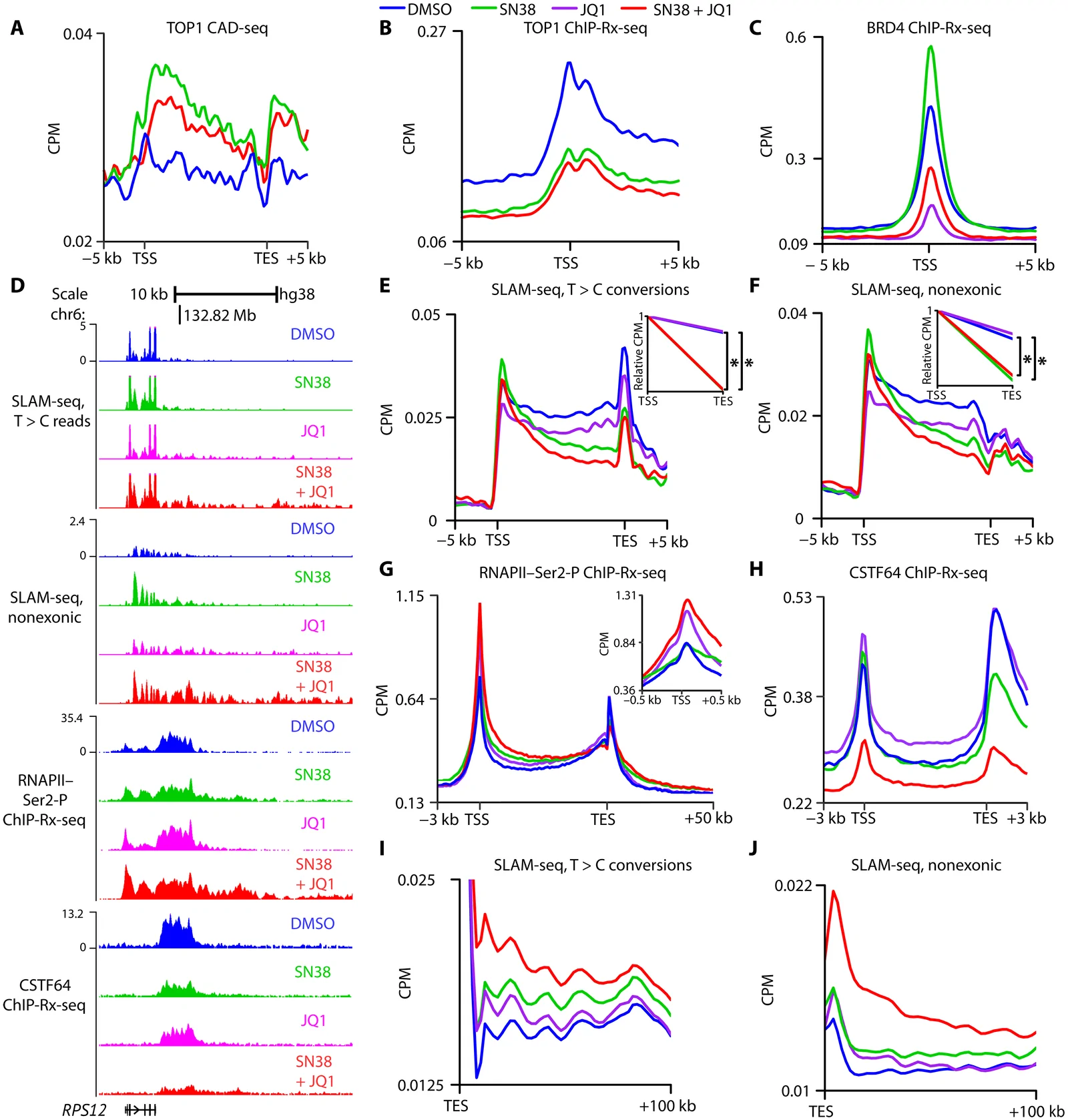
Transcription is synergistically inhibited by the combination treatment SN38 + JQ1. (**A**) Topoisomerase 1 covalent adduct detection sequencing (TOP1 CAD-seq) profile at the 2500 most expressed genes between transcription start site (TSS) and transcription end site (TES) in Bo103 cells treated with dimethyl sulfoxide (DMSO), SN38, or SN38 + JQ1 for 1 hour. Data represented as count per million reads (CPM). Average of biological duplicates. (**B**) TOP1 occupancy at the TSS of the 2500 most expressed genes (±5 kb) in Bo103 cells after 1 hour of treatment. Average of biological duplicates. (**C**) Bromodomain-containing protein 4 (BRD4) occupancy at the TSS of the 10,000 most expressed genes (±5 kb) in Bo103 cells after 4 hours. Average of biological duplicates. (**D**) Genome Browser tracts of (SH)–linked alkylation for the metabolic sequencing of RNA (SLAM-seq) T > C conversion, nonexonic SLAM-seq, RNA polymerase II (RNAPII)–Ser2-P, and CSTF64 chromatin immunoprecipitation sequencing with spike-in control (ChIP-Rx-seq) reads along the gene body and downstream of the gene *RPS12* for all treatment conditions after 4 hours. (**E** and **F**) SLAM-seq reads containing T > C conversions (E) and SLAM-seq nonexonic reads (F) from Bo103 cells plotted between TSS and TES of the 10% longest protein-coding genes after 4 hours of treatment. Inset shows the gradient of the linear regression between TSS and TES (SLAM and NERD indices, respectively; DMSO and JQ1 overlap, SN38 and SN38 + JQ1 overlap). Average of biological triplicates. *P < 0.05 for DMSO versus SN38 and DMSO versus SN38 + JQ1 comparisons, Student’s *t* test. (**G**) RNAPII–Ser2-P occupancy at the 10,000 most expressed genes in Bo103 cells after 4 hours of treatment. Inset shows the distribution of RNAPII–Ser2-P around TSS. Average of biological duplicates. (**H**) CSTF64 occupancy at the 10,000 most expressed genes in Bo103 cells after 4 hours of treatment. Average of biological duplicates. (**I** and **J**) SLAM-seq reads containing T > C conversions (I) and SLAM-seq nonexonic reads (J) from Bo103 cells plotted in the 100-kb downstream of the TES of protein-coding genes after 4 hours of treatment. Average of biological triplicates.

To assess the effect of SN38 and JQ1 on TOP1 activity and BRD4 localization, respectively, we performed TOP1 covalent adduct detection sequencing (TOP1 CAD-seq) (*31*, *32*) and BRD4 chromatin immunoprecipitation sequencing with spike-in control (BRD4 ChIP-Rx-seq) (*33*). TOP1 CAD-seq enables quantification of catalytically active TOP1 on the DNA, known as the TOP1 cleavage complex (TOP1cc). This complex is extremely transient in cells, unless stabilized with TOP1 poisons and inhibitors of proteasomal degradation (*34*). Bo103 cells were treated with dimethyl sulfoxide (DMSO), SN38, or SN38 + JQ1 for 1 hour. For the final 30 min, the proteasome inhibitor MG132 was added to all conditions to inhibit degradation of the TOP1ccs (*35*). SN38 treatment resulted in increased detection of TOP1cc downstream of the transcription start site (TSS) where the phosphorylated CTD of RNAPII stimulates TOP1 activity relative to the untreated condition (Fig. 2A) (*14*). As expected, the appearance of TOP1ccs was proportional to the level of gene expression due to the greater requirement of highly expressed genes for supercoil relief (fig. S2A). Notably, ChIP-Rx-seq for TOP1 demonstrated a profound decrease in TOP1 binding to the TSS after SN38 treatment (Fig. 2B), illustrating how retention of TOP1 on the DNA prevents recruitment of TOP1 to the promoter, likely due to proteasomal degradation of TOP1ccs. The TOP1 CAD-seq data also revealed proportionally fewer TOP1ccs toward the transcription end site (TES) as compared to the TOP1cc profile measured across the genes upon shorter (4 min) irinotecan analog camptothecin treatment (*14*). This likely happens because TOP1 inhibition blocks RNAPII elongation (*36*), leading to fewer TOP1ccs engaged across the gene body. Upon treatment with SN38 + JQ1, the portion of TOP1ccs covalently engaged with the DNA downstream of the TSS was reduced, as compared to SN38 alone (Fig. 2A and fig. S2A), likely reflecting the reduced recruitment of TOP1 (Fig. 2B) due to inhibition of transcription induced by the JQ1 treatment (*37*). BRD4 ChIP-Rx-seq showed that while SN38 increased BRD4 at the TSS likely due to trapped TOP1 blocking BRD4 release from the promoter, JQ1 alone or in combination with SN38 reduced BRD4 occupancy (Fig. 2C). Because BRD4 and TOP1 are key factors in the regulation of promoter-enhancer activity (*38*, *39*), their loss is expected to severely affect enhancer function, as measured by the active enhancer and promoter marker H3 lysine-27 acetylation (H3K27Ac). As expected, H3K27Ac ChIP-Rx-seq revealed a profound decrease in lysine-27 acetylation only upon combination treatment (fig. S2, B and C). This decrease was evident both at enhancers and at promoters, indicating that SN38 + JQ1 incubation globally impairs transcription.

Our model (Fig. 1A) predicts that inhibiting BRD4 recruitment on chromatin and TOP1 stimulation will prevent RNAPII pause release, thus impairing RNAPII progression. If true, then this would result in the accumulation of nascent RNAs directly downstream of promoters and a reduced amount of RNAs at the end of the genes. To test this hypothesis, we directly labeled nascent transcripts by Thiol (SH)–linked alkylation for the metabolic sequencing of RNA (SLAM-seq) (*40*), modifying the protocol to perform the step of retrotranscription with random primers instead of oligo(dT) primers to enable the mapping of nascent RNAs across the entire gene product rather than only at the 3′ end of polyadenylated transcripts. Upon 4 hours of SN38 treatment, the profile of nascent transcripts (i.e., only reads containing T > C conversions from the SLAM-seq dataset) paralleled the TOP1 CAD-seq (Fig. 2A) showing a buildup of transcripts at the beginning of long genes—where the transcription defects would be most apparent—with a depletion of RNAs toward the 3′ ends (Fig. 2, D and E, and table S2). The extent of this change can be quantified using the gradient of the linear regression of the read distribution, which we term the SLAM index (Fig. 2E, inset). Although the index after JQ1 treatment was similar to DMSO, we found an overall reduction in nascent transcripts across the entire gene unit, suggesting defects in RNAPII initiation or elongation. This was possibly due to inhibition of BRD4 bromodomain interaction with acetylated histones at promoters of genes (*37*). The SN38 + JQ1 treatment displayed both a decreased SLAM index and a reduced number of nascent transcripts, indicating that RNAPII transcription was targeted independently through both pathways (Fig. 1A).

We wanted to develop a method to observe nascent transcription from the in vivo samples where labeling transcripts with modified nucleotides would not be possible. We reasoned that since introns are rapidly excised and degraded during cotranscriptional splicing (*41*), plotting only nonexonic reads from the SLAM-seq dataset would allow for bona fide determination of nascent transcription. The latter method exhibited the same reads distribution as the standard SLAM-seq approach (i.e., plotting reads containing T > C conversions; Fig. 2F and fig. S2D) and allowed a similar calculation of the gradient of the nonexonic read distribution, which we term the NERD index (Fig. 2F, inset). Broadly, both methods provided a good approximation for measuring nascent transcription and differed only with respect to the signal at the TES (compare Fig. 2, E and F). The peak observed at the TES from the T > C conversions analysis is due to the enrichment of exons and long 3′ untranslated regions at the TES that makes up a substantial proportion of the SLAM-seq T > C reads, while being excluded from the plot showing nonexonic reads. Thus, our data indicate that analysis of nonexonic read distribution is a viable method for measuring nascent transcription and detecting RNAPII elongation defects.

Last, analysis of the total exonic reads of the SLAM-seq to capture steady-state RNA transcript levels revealed that the SN38 + JQ1 treatment also exhibits more differentially expressed genes relative to individually treated samples (fig. S2E), with “transcription by RNAPII” and “regulation of gene expression” among the top down-regulated gene ontologies, indicating that the cells might undergo reprogramming to reduce overall transcription (fig. S2F). Thus, the SN38 + JQ1 seemed to affect transcriptional pause release and subsequent elongation, as predicted, resulting in stronger cellular response than each individual drug.

### Transcription termination is synergistically inhibited by SN38 + JQ1 treatment

BRD4 controls RNAPII transcription by modulation of RNAPII CTD phosphorylation and TTF recruitment (*42*, *43*). Given the loss of promoter-bound BRD4 observed with JQ1 alone or in combination with SN38 (Fig. 2C), that would lead to an altered pattern of RNAPII–Ser-2 phosphorylation (Ser2-P) and reduced TTF engagement (*18*). ChIP-Rx-seq of RNAPII–Ser2-P upon JQ1 or SN38 + JQ1 treatment showed an increase in Ser2-P levels at promoter-proximal regions (Fig. 2G), which remained after normalization to total RNAPII (fig. S2, G and H), similar to previous findings upon BET protein degradation (*18*). This might represent a futile attempt of the system to compensate for the absence of pause release regulation, by releasing stored PTEF-b (*44*) and delivering it to the RNAPII (*45*). The normalized RNAPII–Ser2-P data also revealed that after SN38 and SN38 + JQ1 treatment, a considerably greater proportion of the total RNAPII was Ser2-phosphorylated across the whole gene despite reduced recruitment to the TSS (fig. S2, G and H). This supports the concept that elongating RNAPII is retained on the DNA for longer likely because of the accumulation of supercoiling not properly removed by TOP1, leading to transcription impairment. Unexpectedly, the RNAPII–Ser2-P signal also extended far downstream of the TES after SN38 + JQ1 when compared to the untreated control, suggesting potential defects in transcription termination (Fig. 2, D and G). In accordance, ChIP-Rx-seq showed that the binding of the 3′ RNA processing factor CSTF64 at both the TSS and TES was markedly reduced after SN38 + JQ1 treatment (Fig. 2, D and H).

Aberrant recruitment of 3′ RNA processing factors has been associated with transcription readthrough of RNAPII downstream the 3′ end of genes (*18*). Analysis of the SLAM-seq revealed that SN38 + JQ1 led to an increase in RNA signal downstream of the 3′ end extending even beyond 100 kb (Fig. 2I). Once again, these results were reproducible by plotting the distribution of nonexonic reads (Fig. 2J). These downstream-of-gene (DoG) transcripts were clearly elevated after the combination treatment relative to all other conditions (fig. S2I). Together, these data suggest that SN38 + JQ1 treatment acts synergistically to impair transcription, while paradoxically leading to readthrough transcription far downstream of the TES due to loss of recruitment of termination factors.

### Genes exhibiting readthrough transcription are highly expressed and heavily paused

**Fig. 3.**
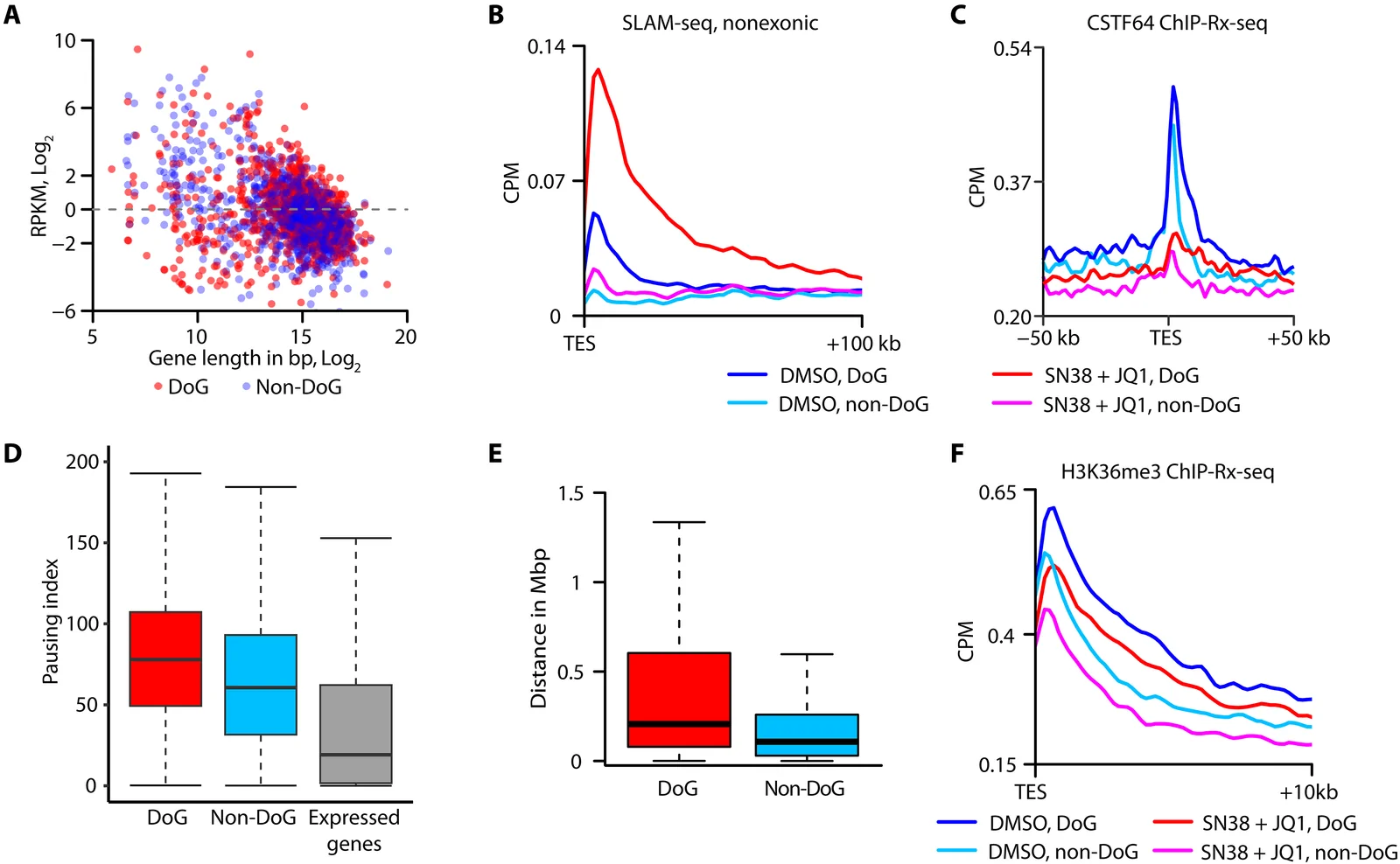
Readthrough transcription is associated with high levels of expression and RNA polymerase II (RNAPII) pausing. (**A**) Comparison of expression level as reads per kilobase per million (RPKM) and gene length of detected genes producing downstream-of-gene (DoG) transcript after SN38 + JQ1 treatment and the generated list of non-DoGs. (**B**) Nonexonic (SH)–linked alkylation for the metabolic sequencing of RNA (SLAM-seq) reads plotted for the 100-kb region downstream of the transcription end site (TES) of DoG and non-DoG genes in untreated [dimethyl sulfoxide (DMSO)] or SN38 + JQ1–treated (4 hours) Bo103 cells. Average of biological triplicates. (**C**) CSTF64 occupancy around TES of DoG and non-DoG genes in Bo103 cells after 4 hours. Average of biological duplicates. (**D**) Boxplot showing RNAPII pausing index of DoG, non-DoG, and all expressed genes. Whiskers indicate lowest and highest values no further than 1.5× interquartile range. (**E**) Boxplot showing the distance from the TES of high-stringency DoGs and non-DoGs to the next expressed gene. Whiskers indicate lowest and highest values no further than 1.5× interquartile range; outliers are excluded. (**F**) H3K36me3 occupancy 10-kb downstream of the TES of DoG and non-DoG genes in Bo103 cells after 4 hours of treatment with DMSO or SN38 + JQ1. Average of biological duplicates.

Although CSTF64 binding was reduced in all genes after SN38 + JQ1 treatment, DoG transcription was only detected for a subset of genes. To further characterize why DoG genes are vulnerable to readthrough transcription, we generated a set of non-DoG genes with similar length and expression levels for comparison, assuming that they undergo the same topological challenges as the DoG genes (Fig. 3A). These DoG and non-DoG gene sets contained 921 and 867 genes, respectively. Analysis of the SLAM-seq read downstream of the TES demonstrates that even under untreated conditions, DoG genes exhibited higher levels of readthrough transcription compared to the non-DoG genes (Fig. 3B), suggesting that DoG genes are prone to readthrough transcription even at a basal state. This effect was accentuated after SN38 + JQ1 exposure (Fig. 3B). Because of their dependency on TOP1 activity and BRD4 regulation, we surmised that genes undergoing readthrough transcription must be highly expressed and highly paused. We observed that most genes exhibiting readthrough transcription were characterized on average by higher levels of expression in comparison to all expressed genes (fig. S3A). In addition, while comparison of these two gene sets demonstrated that both have a similar extent of CSTF64 loss after SN38 + JQ1 treatment (Fig. 3C), DoG genes have a higher pausing index than non-DoG genes based on our total RNAPII ChIP-Rx-seq data (Fig. 3D). Moreover, the DoG genes exhibited higher levels of RNAPII-Ser-2P than non-DoG genes, suggesting stronger dependence on RNAPII CTD modifications (fig. S3B).

If susceptibility to readthrough transcription originates from dysregulation of RNAPII modifications at the pausing site where TOP1 and BRD4 are functional, then readthrough would not be detected until the RNAPII had transcribed the length of the gene. A quantitative polymerase chain reaction (qPCR) time-course experiment demonstrated that short genes such as *SERP1* (4.5 kb) and *SEC61B* (8.3 kb) showed readthrough transcription after 1 hour, while the longer gene *BPNT2* (36 kb) did not exhibit readthrough transcription until 2 hours after treatment with SN38 + JQ1, despite the primers being equidistant from the respective TESs (fig. S3C). In addition, considering the average RNAPII transcription rate in the presence of TOP1 poison camptothecin is 1 kb/min (*46*), genes longer than approximately 240 kb should not exhibit readthrough transcription after 4 hours of treatment. DoG genes were typically shorter than 150 kb in length (fig. S3D). These data support the concept that RNAPII is primed to undergo readthrough transcription at the pausing site as opposed to the TES.

We reasoned that the readthrough transcription that extends hundreds of kilobase downstream the TES would likely disturb other chromatin-related processes. To this end, we curated a “high-stringency” list of DoG genes characterized by elevated transcription of 30- to 45-kb downstream of the TES after SN38 + JQ1 treatment relative to untreated control. This subset was even more specific for SN38 + JQ1, as the individual treatments had fewer DoG genes that matched these criteria (fig. S3E), and readthrough transcription could be detected up to 200-kb downstream of the TES (fig. S3F). To understand how this readthrough transcription may affect other intergenic or intragenic regions, we calculated the distance of the closest expressed gene downstream of the high-stringency DoG genes. Readthrough transcription from these high-stringency genes was more likely to extend into gene-free regions relative to a non-DoG gene set of similar expression and length distribution as evident by the average higher distance to the next expressed gene located downstream (Fig. 3E).

### Readthrough transcription affects repressive chromatin

If DoG transcripts are more likely to extend into gene free regions, their chromatin must be silenced to safeguard transcription fidelity. Lysine-36 of histone 3 is typically trimethylated (H3K36me3) by SET domain containing 2, histone lysine methyltransferase (SETD2) following RNAPII passage to prevent spurious transcription (*47*–*49*). In addition, loss of SETD2 is associated with increased readthrough transcription, suggesting that H3K36me3 plays a role in blocking DoG transcripts (*50*). As SN38 + JQ1 treatment was found to reduce SETD2 expression (table S2) and SETD2 is the sole methyltransferase responsible for H3K36me3, we investigated this chromatin modification by ChIP-Rx-seq. Under untreated conditions, H3K36me3 levels were higher in the 10-kb region downstream of the 3′ ends of DoG versus non-DoG genes (Fig. 3F, compare dark blue and light blue curves), suggesting that different genes show a differential susceptibility to DoG transcription despite similar loss in CSTF64 binding. In addition, treatment with SN38 + JQ1 overall reduced the amount of the H3K36me3 marker showing how induction of readthrough transcription by SN38 + JQ1 treatment modulates the local chromatin environment. Thus, the combined inhibition of TOP1 and BRD4 promotes changes in the chromatin structure, DoGs, toward a more derepressed state.

### SN38 + JQ1–driven readthrough transcription affects late replication causing potential replication-transcription collisions

**Fig. 4.**
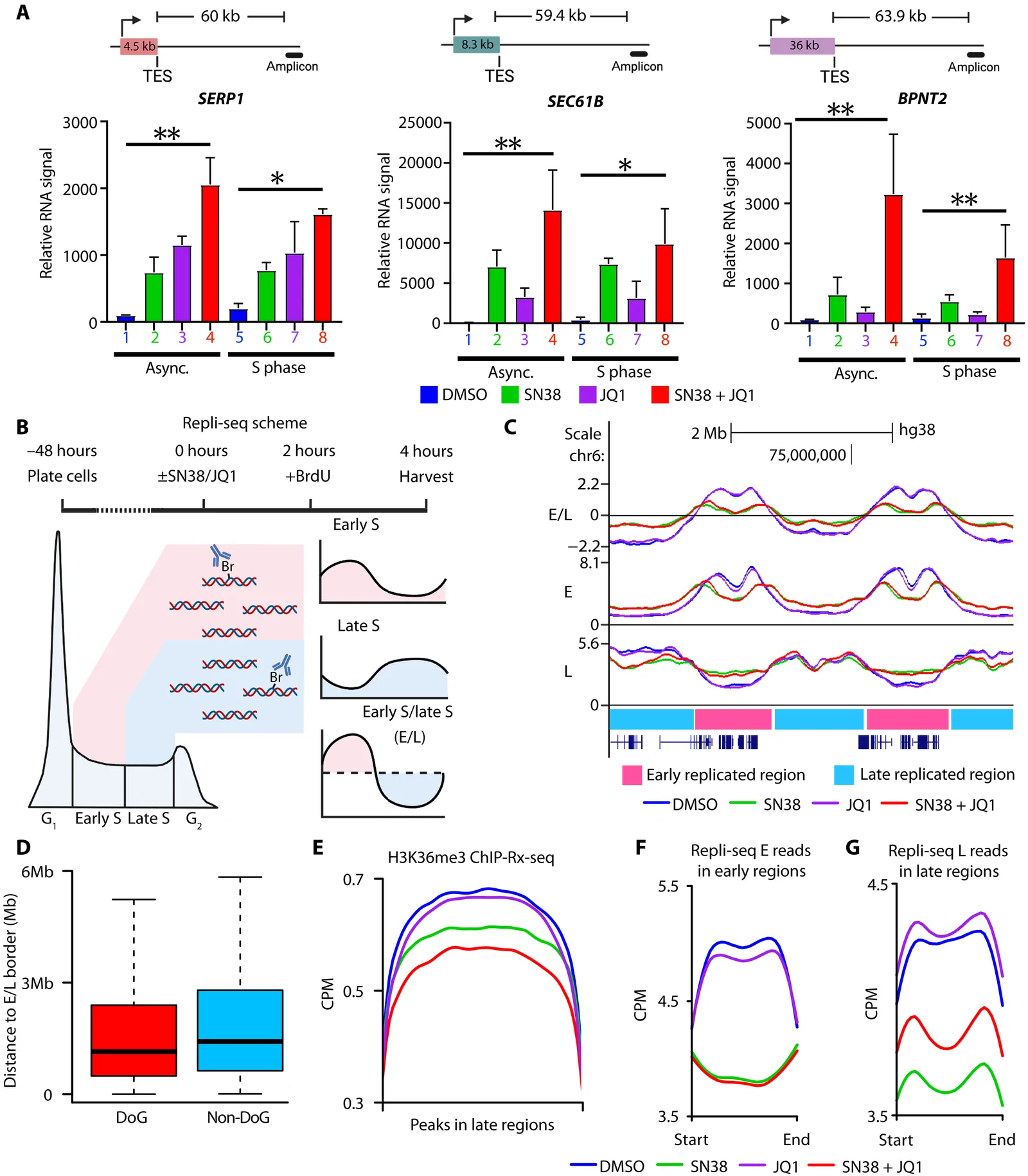
Readthrough transcription induced by SN38 + JQ1 enhances firing of dormant origins by chromatin decompaction. (**A**) Readthrough transcription persists in S phase upon treatment with SN38 + JQ1, as detected at selected region downstream of the downstream-of-gene (DoG) genes *SERP1*, *SEC61B*, and *BPNT2* after 4 hours [*n* = 3, relative to dimethyl sulfoxide (DMSO) control; error bars represent SD]. *P < 0.05 and **P < 0.01, Student’s *t* test. (**B**) Scheme of Repli-seq. The approach allows for determination of early and late replicated regions in the genome. (**C**) Example Genome Browser tracks of replication timing (RT) (E/L), early (E), and late (L) replication upon Bo103 cell treatment with DMSO, SN38, JQ1, or SN38 + JQ1. Early (pink) and late (light blue) replicated regions are denoted by colored boxes underneath the tracks. Average of biological duplicates. (**D**) Boxplot showing the distance downstream from the transcription end site (TES) of DoGs and non-DoGs in early replicated regions to the next early-to-late border (*P* < 0.0005). Whiskers indicate lowest and highest values no further than 1.5× interquartile range; outliers are excluded. (**E**) H3K36me3 peaks in late replicated regions in Bo103 cells treated with DMSO, SN38, JQ1, or SN38 + JQ1 for 4 hours. Average of biological duplicates. (**F**) Repli-seq E read coverage at early replicated regions. Average of biological duplicates. (**G**) Repli-seq L read coverage at late replicated regions. Average of biological duplicates.

If the transcriptionally engaged RNAPII can continue transcribing into intergenic regions, thus remodeling the chromatin in those regions, they might potentially affect the translocation of other DNA revolving machineries, such as replisomes. To test this hypothesis, we first verified whether readthrough transcription is detectable during S phase upon SN38 + JQ1 treatment and then whether it affects replication fork progression. Cells were synchronized in S phase with the DNA synthesis inhibitor hydroxyurea (*51*), released into fresh medium and treated with SN38 or JQ1 alone or in combination for 4 hours (fig. S4A). While, under untreated conditions, the RNA signal at the analyzed DoG regions was negligible, it increased remarkably upon drug treatment reaching its maximum with SN38 + JQ1 (Fig. 4A, compare lane 5 versus lane 8). We detected comparable DoG transcripts between S phase–synchronized and –asynchronous cells (Fig. 4A, compare lane 4 versus lane 8), indicating that SN38 + JQ1–driven readthrough transcription persists during S phase.

Next, we applied Repli-seq (*52*) to observe changes in replication timing (RT) after 4 hours of treatment with SN38 or JQ1, alone or in combination. Briefly, replicating cellular DNA was labeled by 5-bromo-2′-deoxyuridine (BrdU) pulsing, cells were then harvested and sorted into early (E) and late (L) S phase populations. BrdU-labeled DNA was then immunoprecipitated and sequenced to determine the RT of each genetic region (Fig. 4B). The RT (i.e., the regions referred to as early or late replicating based on the E/L profile) did not broadly change between treatment conditions, although the reduced signal under both SN38 and SN38 + JQ1 conditions indicated that TOP1 inhibition greatly affects the overall replication rates (Fig. 4C), as expected, given the requirement of TOP1 for proper replication fork progression (*53*).

We predicted that the readthrough RNAPIIs induced by SN38 + JQ1 treatment may proceed across replication boundaries, thereby interfering with replication origin firing and fork progression. We divided the genome into early and late replicating regions based on the Repli-seq signal using the RepliScan algorithm (*54*). We found that 15% of DoG transcripts were in late replicating regions, while 85% of DoG transcripts were in early replicating regions. Notably, a few of the latter DoG transcripts were upstream of replication boundaries, with 8.2% (55 genes) within 200 kb of the early-to-late regions. Considering that SN38 + JQ1 caused DoG transcription 200 kb beyond the TES (fig. S3F), this suggests that readthrough transcription can continue through replication boundaries. The distance between DoG genes and early-to-late replication boundaries was markedly smaller than seen with non-DoG genes (Fig. 4D), and the direction of readthrough transcription typically travels from early to late replication regions (fig. S4B).

If readthrough transcription occurs in heterochromatic late replicating regions or at least in regions where transcription is typically repressed, then it might affect chromatin compaction, leading to a derepressed chromatin state. The levels of H3K36me3 in late regions were reduced when comparing untreated and SN38 + JQ1–treated samples (Fig. 4E). This change in chromatin status could, in turn, provoke activation of normally dormant origins, which usually do not fire under untreated conditions, as it is more likely that the replication fork originating from a more active origin reaches them beforehand. However, under conditions where replication is globally affected (e.g., SN38 treatment), dormant origins might get activated as previously seen (*55*). We quantified the E and L Repli-seq reads in the early and late replicating regions (Fig. 4C, pink and blue boxes), respectively. In early replicating regions, both SN38 and SN38 + JQ1 treatments showed an equal reduction in DNA replication, suggesting that SN38 + JQ1 treatment had no additional effect on early replication (Fig. 4F). In stark contrast, SN38 + JQ1 treatment partially rescued replication in late replicating regions as increased signal could be measured relative to SN38 alone (Fig. 4G, compare red and green curves). Thus, the data support our hypothesis that SN38 + JQ1–driven readthrough transcription induces dormant origin firing in late replicated areas, specifically under conditions when early replication elongation is impaired by SN38.

### SN38 + JQ1 treatment induces DNA damage in S phase and cell stress signaling in G_1_ and G_2_ phases

TOP1 activity is required to resolve replication-dependent supercoiling during S phase (*24*). Upon TOP1 inhibition, TOP1 is trapped on the DNA resulting in DNA double-stranded breaks during S phase by replication run-off (*56*, *57*). Furthermore, loss of TOP1 activity has been shown to inhibit replication fork progression and to induce replication-transcription interference likely due to supercoiling accumulation (*53*, *58*–*60*). This, in turn, can lead to cell cycle arrest and cell death (*61*, *62*). We predicted that SN38 + JQ1–induced transcription into gene-free regions and late replication origin firing would increase the probability of replication fork stalling and DNA damage compared to TOP1 inhibition by SN38 alone.

**Fig. 5.**
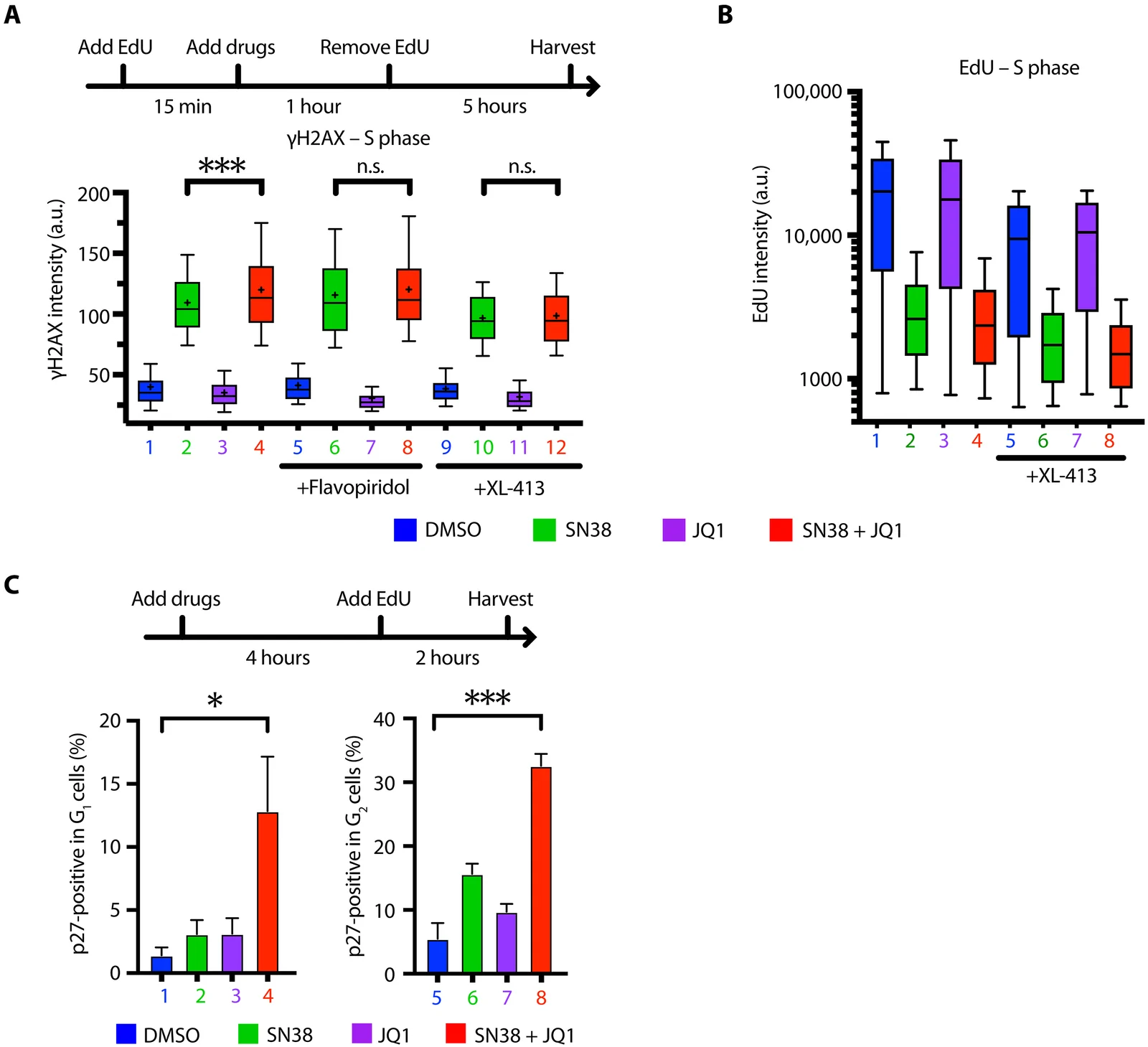
SN38 + JQ1 treatment induces readthrough transcription-dependent replication stress in S phase and cell stress signaling in G_1_ and G_2_ phases. (**A**) Top: Schematic of treatment. Bottom: Immunofluorescence quantitation of γH2AX intensity in S phase (EdU-positive) upon dimethyl sulfoxide (DMSO), SN38, JQ1, or SN38 + JQ1 treatment ± 2 μM flavopiridol or 15 μM XL-413 for 6 hours. Mean represented by +, whiskers extend to 10 to 90%. a.u., arbitrary units. Representative plot of *n* = 3. ***P < 0.001, Student’s *t* test. n.s., not significant. (**B**) Flow cytometry analysis of EdU incorporation in S phase cells after DMSO, SN38, JQ1, or SN38 + JQ1 treatment ± 15 μM XL-413 after 6 hours. Whiskers extend to 10 to 90%. Representative plot of *n* = 3. (**C**) Top: Schematic of treatment. Bottom: Flow cytometry quantitation of p27-positive cells in G_1_ and G_2_ phases of the cell cycle upon DMSO, SN38, JQ1, or SN38 + JQ1 treatment ± 2 μM flavopiridol after 6 hours (*n* = 4; error bars represent SD). *P < 0.05 and ***P < 0.001, Student’s *t* test.

To investigate this potential mechanism of drug synergy, we assessed the extent of DNA damage by quantification of DNA damage marker γH2AX (*63*) in S phase cells labeled by 5-ethynyl-2'-deoxyuridine (EdU) incorporation. In agreement with previous research (*24*), γH2AX was specifically up-regulated in EdU^+^ S phase cells in response to SN38 exposure (Fig. 5A, compare lane 1 versus lane 2, and fig. S5A). This signal was significantly increased after SN38 + JQ1 treatment, indicating a synergistic DNA damage response (Fig. 5A, compare lane 2 versus lane 4). We conceived two independent methods to test whether the DNA damage upon SN38 + JQ1 was dependent on transcription interference with the replisome. The CDK9 inhibitor flavopiridol inhibits RNAPII elongation and can block both genomic and readthrough transcription (*64*) in asynchronous Bo103 cells (fig. S5B). On the other hand, the CDC7 inhibitor XL-413 is able to inhibit DNA replication initiation (*65*), as shown by reduced EdU incorporation into replicating DNA independent of SN38 + JQ1 treatment (Fig. 5B, compare lanes 5 to 8 versus lanes 1 to 4). If the increased γH2AX seen upon SN38 + JQ1 treatment stems from the transcription-dependent replication stress, then cotreatment with either flavopiridol or XL-413 should revert SN38 + JQ1–induced γH2AX signaling back to the level of SN38 treatment alone. Although flavopiridol treatment with SN38 alone increased γH2AX, in line with previous reports of flavopiridol potentiating the apoptotic effect of TOP1 poisons (*66*), cotreatment with either flavopiridol (Fig. 5A, compare lane 6 versus lane 8) or XL-413 (Fig. 5A, compare lane 10 versus lane 12) reverted SN38 + JQ1–induced γH2AX back to SN38 levels, demonstrating that these treatments only abrogated the SN38 + JQ1–specific effects.

We next tested whether the SN38 + JQ1 treatment exhibited any synergistic effects in G_1_ and G_2_ cell cycle phases. The tumor suppressor p27 can arrest the cell in G_1_ under stress conditions to prevent potentially genotoxic consequences of DNA replication (*67*). It can also cause G_2_ arrest in response to DNA damage in S phase to avoid mitotic catastrophe (*68*, *69*). Persistent p27-dependent cell cycle arrest is shown to induce apoptosis in many cancer types (*70*). Therefore, we postulated that p27 may become up-regulated in G_1_ and G_2_ in response to replication interference induced by SN38 + JQ1 treatment. We observed a clear synergistic up-regulation of p27 expression in both G_1_ (Fig. 5C, compare lane 1 versus lane 4) and G_2_ (Fig. 5C, compare lane 5 versus lane 8) phases only after exposure to SN38 + JQ1. Overall, these data suggest that the SN38 + JQ1–induced readthrough transcription provokes replication stress and triggers a downstream DNA damage and stress signaling response.

### PDXs remain sensitive to irinotecan + JQ1 treatment upon multiple treatment cycles in vivo

**Fig. 6.**
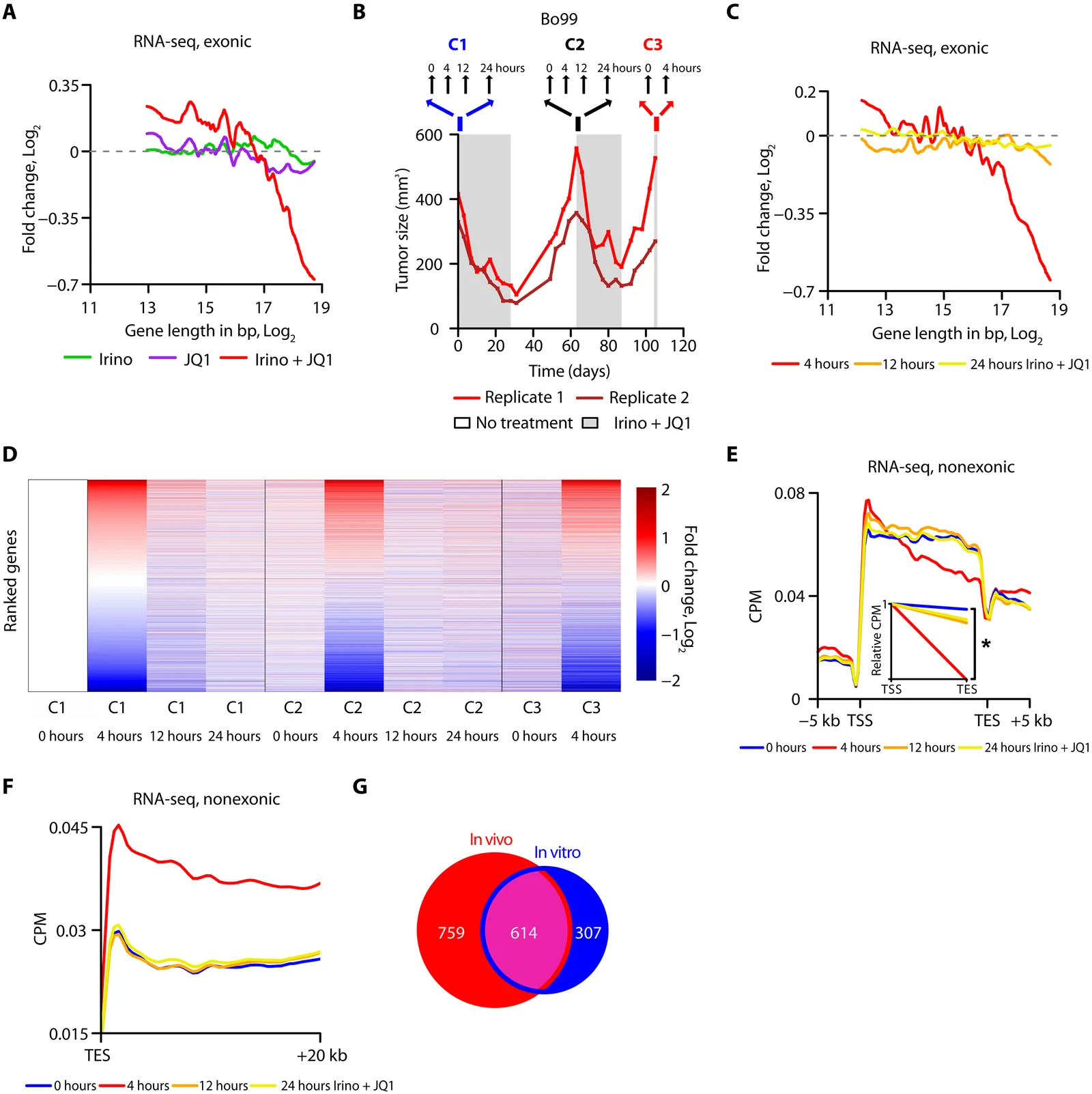
Irinotecan + JQ1 in patient-derived xenografts (PDXs) triggers readthrough transcription and does not show emergent resistance over time. (**A**) Moving average of fold change (log_2_) derived from exonic RNA-seq reads of treated (irinotecan, JQ1, and irinotecan + JQ1) versus untreated Bo99 PDX plotted against the gene length (log_2_). Average of biological duplicates. (**B**) Top: Dosing and harvesting schedule. Bottom: Example growth curve of two Bo99 PDX tumors, subjected to 3 cycles of treatment with irinotecan + JQ1. (**C**) Same as (A), but Bo103 PDX was treated with irinotecan + JQ1 for 4, 12, and 24 hours and compared to untreated Bo103 PDX. Average of three to four tumors per condition. (**D**) Fold change (log_2_) of RNA-seq reads of Bo103 PDX for each time point versus untreated. (**E**) Nonexonic RNA-seq reads of Bo103 PDX plotted between the transcription start site (TSS) and transcription end site (TES) of the 10% longest protein-coding genes. Inset shows the gradient of the linear regression between TSS and TES (NERD index). Average of three to four tumors per condition. *P < 0.05, Student’s *t* test. (**F**) Nonexonic RNA-seq reads of Bo103 PDX plotted in the 20-kb region downstream of the TES of protein-coding genes. Average of three to four tumors per condition. (**G**) Venn diagram of genes producing downstream-of-gene (DoG) transcripts detected in cultured Bo103 cells and in the Bo103 PDX after 4 hours of SN38/irinotecan + JQ1 treatment.

Having established a mechanistic model for the synergism of SN38 + JQ1 in killing tumor cells, we then sought to translate our in vitro finding into preclinical settings to determine whether we observe the same phenotypes in vivo. We performed RNA sequencing (RNA-seq) on the Bo99 PDX tumors described in fig. S1A, treated with irinotecan or JQ1, alone or in combination. Analysis of the data showed a down-regulation of long genes after irinotecan + JQ1 treatment (Fig. 6A and table S3), due to transcription inhibition. This effect was considerably more profound relative to the individual treatments, in contrast to the in vitro results (fig. S6A), suggesting that there may be even stronger synergy in vivo.

To assess the early effects of irinotecan + JQ1 on transcription, we designed the RNA-seq protocol for Bo99 and Bo103 tissue material, performing the step of retrotranscription with random primers. This allowed detection of nonexonic reads and extrapolate information about nascent RNAs. We also devised a treatment scheme in which we administered the PDX carrying mice with multiple cycles of irinotecan + JQ1 [referred to as cycle 1 (C1), cycle 2 (C2), and cycle 3 (C3)], harvesting tumors 0, 4, 12, and 24 hours after the start of each treatment cycle (Fig. 6B). This way also allowed us to investigate whether tumor cells treated with the irinotecan + JQ1 would develop resistance over time (tables S4 and S5). As expected, long genes were strongly down-regulated in response to 4 hours of treatment with irinotecan + JQ1 (fig. S6B), and the tumors regressed upon subsequent treatment cycles (Fig. 6B and fig. S6, C to E), indicating that the cells do not gain resistance to the treatment. Common markers of resistance to either irinotecan or JQ1 were not significantly dysregulated in the C3 0 hour timepoint relative to C1 0 hour timepoint, in agreement with continued drug sensitivity of the tumors to irinotecan + JQ1 (fig. S6, F and G, and table S6). Long gene inhibition was lost 12 and 24 hours after treatment (Fig. 6C), indicating that the transcription inhibition was transient and probably vanished as the drugs were metabolized and excreted. Overall, we found high concordance of the response after each treatment cycle and a general reversion after 12 hours (Fig. 6D).

By plotting the nonexonic reads onto the metagene, we then analyzed how the irinotecan + JQ1 treatment affected transcription. First, we observed that 4-hour combination therapy provokes a buildup of short transcripts around the start of the genes, particularly of long genes, as seen in vitro (Fig. 2, E and F). This accumulation dissipated after 12 and 24 hours (Fig. 6E and fig. S6H). Readthrough transcription was evident 4 hours after irinotecan + JQ1 exposure (Fig. 6F), was found to extend as far as 500 kb (fig. S6I), and was detectable at a highly similar set of genes as seen in vitro (Fig. 6G) with 45% of DoG genes from the in vivo experiment also detected in vitro.

### Irinotecan + JQ1 combination specifically targets transcriptionally addicted tumors over normal cells

Tumor cells are dependent on transcriptional dysregulation to drive oncogenic growth (*2*). This “addiction” to oncogenic drivers of gene expression can render tumor cells susceptible to disruption of transcription. If normal noncancerous cells are less sensitive to transcription inhibition, this treatment would be more selective toward cancer cells and may provide a therapeutic window for clinical intervention. Therefore, if TOP1 and BRD4 inhibition treatment has clinical potential as a transcription targeting regimen, then we would expect it to elicit a substantially reduced response in normal tissues, as we observed in vitro with normal immortalized pancreatic hTERT-HPNE cells (fig. S1F).

**Fig. 7.**
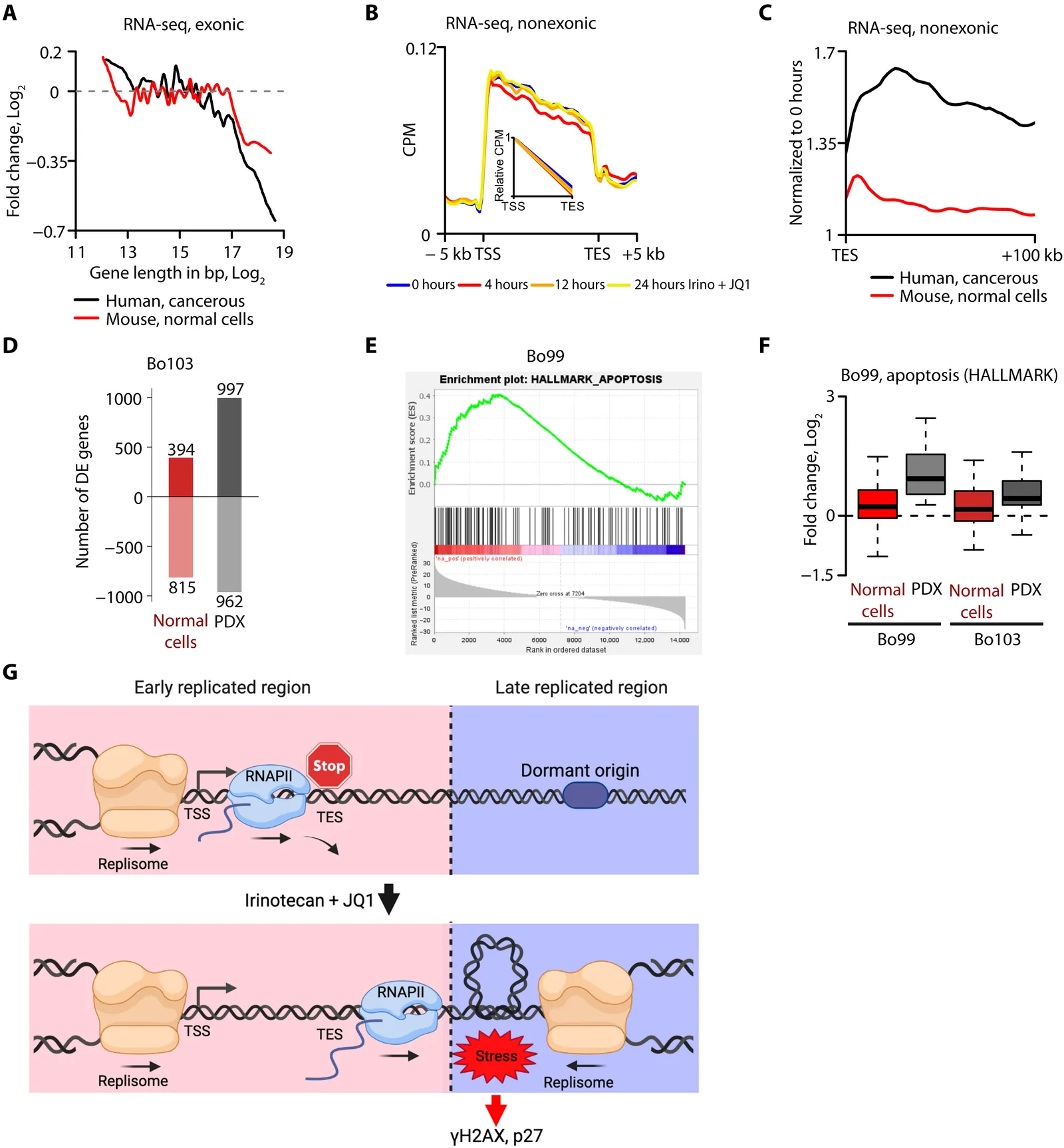
Transcription is preferentially affected in Bo103 patient-derived xenografts (PDXs) upon irinotecan + JQ1 compared to normal mouse cells. (**A**) Moving average of fold change (log_2_) of exonic RNA-seq reads from Bo103 PDX treated with irinotecan + JQ1 for 4 hours and normal mouse cells. Average of four tumors per condition. (**B**) Nonexonic RNA-seq reads from normal mouse cells plotted between the transcription start site (TSS) and transcription end site (TES) of the 10% longest protein-coding genes. Inset shows the gradient of the linear regression between TSS and TES (NERD index). Average of three to four tumors per condition. No significant difference between NERD indexes. (**C**) Nonexonic RNA-seq reads of PDX tumors and normal mouse cells from Bo103 PDXs treated for 4 hours with irinotecan + JQ1. Reads are plotted in the 100-kb region downstream of the TES of protein-coding genes. Data expressed as count per million (CPM) and normalized to the corresponding CPM values at 0 hours. Average of four tumors per condition. (**D**) Statistically significant (adjusted *P* < 0.05) differentially expressed (DE) up- and down-regulated genes in Bo103 PDX tumor and associated mouse normal cells upon irinotecan + JQ1 for 4 hours. (**E**) Gene set enrichment analysis (GSEA) for the gene ontology term apoptosis [normalized enrichment score = 1.50, false discovery rate (FDR) = 0.026] plotting gene enrichment after 4 hours irinotecan + JQ1 of Bo99 PDX tumor. (**F**) Relative expression of the core enriched apoptosis genes from (E) in Bo99 and Bo103 PDX tumors, both human cancer and mouse normal cells. (**G**) Working model. Under untreated conditions, transcription and replication are coordinated with replication initiating in open, highly transcribed regions. Upon SN38/irinotecan + JQ1 treatment, readthrough transcription affects the chromatin state and induces dormant origin firing in late replicated areas. This will interfere with the established replication timing (RT) pattern leading to replication stress, DNA damage, and cell cycle arrest.

Since a subset of infiltrating mouse cells were coharvested with each PDX tumor (6 to 12% in Bo99 and 23 to 38% in Bo103), we could map RNA-seq reads to the mouse genome to understand how irinotecan + JQ1 treatment affected the expression of nascent RNAs in the mouse normal cells (tables S4 and S5). Transcription of long genes was also reduced in mouse normal cells after 4 hours of irinotecan + JQ1 but reverted after 12 and 24 hours, as seen in the human tumor cells (Fig. 7A and fig. S7A), indicating that transcription is targeted in both cell types. However, in contrast to the PDX cancer cells (Fig. 6E and fig. S6H), the NERD index of the normal cells showed no substantial changes in the distribution of reads across the gene body after the different treatments (4 hours), either when observing all genes (fig. S7B) or long genes sensitive to elongation inhibition (Fig. 7B). Together, these data imply that while transcription elongation is inhibited also in the normal cells, the absence of short transcripts accumulating immediately downstream of the TSS suggests that transcription does not continue to be initiated and subsequently stalled by trapped TOP1. Most notably, readthrough transcription was barely detectable in the nonmalignant relative to the malignant cells (Fig. 7C).

That the normal mouse tissue was not affected in promoter pausing regulation by irinotecan + JQ1 indicated that (i) there should be fewer differentially expressed genes relative to the PDX tissues treated with the same drugs and (ii) signaling pathways involved in cellular stress should not be activated upon treatment. As expected, there were notably fewer differentially expressed genes in the normal compared to the PDX tissue (Fig. 7D and fig. S7C). While the number of down-regulated genes in PDX and normal samples is more similar in the Bo103 PDX due to inhibition of long genes (Fig. 7A), the number of up-regulated genes was significantly greater in the PDX tissue, indicating a stronger response to the treatment. Furthermore, gene set enrichment analysis (GSEA) (*71*) revealed that gene ontologies associated with cell death were not enriched in the mouse normal cells. The core genes from the hallmark gene ontology term “apoptosis” were consistently up-regulated in both Bo99 and Bo103 PDXs, but the homologous mouse genes in coharvested normal mouse cells were not affected (Fig. 7, E to F). Together, these results suggest that targeting transcription via dual inhibition of TOP1 and BRD4 can specifically target KRAS- and MYC-driven pancreatic tumors and provide viable and clinically applicable therapy for hard-to-treat cancer entities such as PDAC and panNEC.

## DISCUSSION

While there have been recent advances made toward directly targeting KRAS^G12^ mutant tumors (*72*, *73*), selective targeting of oncogenic RAS activity is challenging, and early clinical trials have been short lived (*74*, *75*). Therefore, many strategies are directed to target KRAS downstream signaling to indirectly block the oncoprotein’s effect (*76*). Here, we show that combining BET inhibition with irinotecan to indirectly target oncogenic signaling is able to induce tumor regression, with two of the three PDAC PDX models tested reaching partial response, including one model of the difficult-to-treat basal (or quasi mesenchymal) subtype. The panNEC PDX model responded unexpectedly well with complete response, suggesting that this combination could be highly effective for both types of pancreatic cancer to be explored in future clinical studies. Considering the robust inhibition of KRAS-driven pancreatic tumors reported here, we propose that directly targeting transcription should be considered as a promising treatment strategy in clinical research.

SN38 + JQ1 treatment promotes transcription downstream of the termination site by making the RNAPII not competent for interaction with TTFs downstream of the pausing site. Acute degradation of BRD4 has previously been shown to prevent TTF recruitment and efficient transcription termination, although JQ1 treatment alone was insufficient to cause extensive readthrough (*18*). Although JQ1 and SN38 + JQ1 had similar effects on BRD4 displacement on chromatin (Fig. 2C), only the combination treatment caused profound loss of TTF recruitment and elevated readthrough transcription (Fig. 2, H to J), suggesting that loss of BRD4 binding cannot completely account for the readthrough transcription phenotype. The mechanism by which coinhibition of BRD4 and TOP1 can synergistically prevent TTF recruitment remains unclear, particularly since the individual treatments showed a mild increase in TTF CSTF64 at the TSS (Fig. 2H). One possibility may involve the independent effects of both drugs on RNAPII phosphorylation. TOP1 poisoning by camptothecin is known to release PTEF-b from its inactive complex with 7SK small nuclear ribonucleoprotein resulting in RNAPII hyperphosphorylation (*44*). In addition, in the absence of BRD4, RNAPII can be hyperphosphorylated through PTEF-b by the super elongating complex (SEC) (*45*, *77*). The SN38 + JQ1 treatment therefore would be expected to induce RNAPII hyperphosphorylation via two separate mechanisms: the release and activation of stored PTEF-b and the phosphorylation via SEC rather than BRD4. Our RNAPII–Ser-2 ChIP-Rx-seq data (Fig. 2G) highlight a JQ1-dependent increase in Ser-2–phosphorylated RNAPII at the TSS and a SN38-dependent increase toward the 5′ of the gene, with the SN38 + JQ1 combination exhibiting both phenotypes. It is possible that the hyperphosphorylated state of RNAPII, along with the switch toward SEC-dependent activation preventing BRD4-dependent recruitment of TTFs, drives readthrough transcription. The profound effects of flavopiridol, a potent inhibitor of PTEF-b (*78*), on readthrough transcription strongly suggest that the observed effect is dependent on PTEF-b activation.

By demonstrating that readthrough is indeed sufficient to induce stress and causes both cell cycle arrest and apoptosis in transcriptionally addicted cancer cells (Fig. 7G), this study reveals the potential of inducing readthrough transcription to treat certain types of cancers. The differential response we present here between highly malignant human cancer cells and normal mouse cells suggests that targeting transcription and inducing readthrough transcription by BRD4 + TOP1 coinhibition may be particularly effective in transcriptionally addicted malignant cells. It thus represents a promising and clinically feasible treatment option, not only for the hard-to-treat PDAC and panNEC but potentially also for other solid tumor types.

Cancer cells require high levels of transcription and replication to maintain oncogenic proliferation. Cell transformation can induce replication stress, subsequently leading to DNA damage, senescence, and cell death (*79*, *80*). Thus, to continue proliferating, cancer cells must adapt to tightly regulate the coordination between the replication and transcription machineries. This makes tumor cells susceptible to targeting replication stress therapeutically (*81*). The importance of TOP1 and BRD4 to maintain optimal transcription is well established. However, both proteins are also involved in regulation of DNA replication. TOP1 is essential for removing supercoils that accumulate in response of replisome translocation (*82*), while BRD4 regulates DNA replication checkpoint signaling (*83*). Therefore, characterization of the synergistic effect of TOP1 + BRD4 cotargeting must account for the independent roles of replication and transcription and how these processes interact. We show that replication stress in response to SN38 + JQ1 treatment is dependent on both dysregulated transcription and replication dormant origin firing (Fig. 5A), highlighting the dual targeting of this combination therapy.

There has been a resurgence of interest in readthrough transcription over the past 10 years (*84*). This process can be triggered by heat or osmotic shock or by viral infection (*64*, *85*). It has been described to be driven by loss of termination factors, as we demonstrate in our study, or by disruption of other RNA processing factors such as Integrator (*86*). The purposes and outcomes of readthrough transcription are still being elucidated. Some reports place this process downstream of stress signaling to protect the cell from external stresses. For example, long noncoding RNA produced by readthrough transcription can act as a nuclear scaffold to maintain nuclear integrity after osmotic stress (*87*). However, in other instances such as during influenza A virus (IAV) infection, readthrough transcription can directly generate a stress response by remodeling the three-dimensional (3D) structure of the genome (*64*). In this regard, the Influenza virus NS1A binding protein (NS1) induces transcriptional readthrough, which continues through topologically associated domain (TAD) boundaries. This displaces cohesin from TAD boundaries and decompacts the heterochromatic DNA region to create a more permissive chromatin (*64*). Here, we demonstrate that readthrough transcription driven by SN38 + JQ1 treatment is frequently proximal to replication boundaries, which are largely congruous with TADs (*88*), and exhibits many phenotypes consistent with permissive heterochromatin including loss of the silencing histone marker H3K36me3 and dormant origin firing in late S phase replication. IAV infection has also been demonstrated to up-regulate the stress response protein p27 (*89*), supporting our data (Fig. 5C). Furthermore, the elevated replication in heterochromatic compartments was not strictly isolated to regions of readthrough transcription, suggesting that local chromatin decompaction at DoG transcription sites can lead to broad decondensation of late replicating heterochromatin. Recent research into heterochromatin maintenance through phase separation (*90*, *91*) may suggest that readthrough transcription could disrupt the entire phase-separated compartment, thus propagating chromatin compartment switching at a distance.

Further evidence supporting the concept that the SN38 + JQ1 response phenotype is related to aberrant transcription can be extrapolated from our earlier work. We previously showed that the colon carcinoma HCT116KI cell line, which has an exon 4 deletion in *TOP1* preventing RNAPII-TOP1 interaction, without affecting the enzymatic activity of TOP1, exhibited greatly reduced synergy between SN38 and JQ1 compared to the isogenic wild-type cell line (*14*). While these cancer cells showed clear perturbation in transcription through loss of coordination between transcription and supercoil relief, they are equally sensitive as wild-type cells to inhibition of replication by fluorouracil, suggesting that replication is not impaired in the HCT116KI cells (*92*).

Another potential advantage of directly targeting oncogenic transcription is the likely reduced chance of resistance development upon repeated treatment cycles. Precision medicine, the therapeutic targeting of specific drivers of cancer, can initially elicit rapid tumor regression, but subpopulations of tumors emerge driven by alternative pathways that are resistant to continued therapy (*93*). However, in contrast to the redundancy of signaling pathways, transcription is an essential and irreplaceable feature of cellular homeostasis. Our work indicates that the downstream response to the drugs with regard to readthrough transcription and stress signaling is profoundly elevated in the PDX-containing malignant cells relative to the normal mouse cells from the same lesion (Fig. 7). Since cancer cells are “addicted” to elevated transcription rates, the direct targeting of oncogenic transcription provides a therapeutic window for precision treatment.

This study was limited to characterizing the response to TOP1 and BRD4 inhibition both in vitro by cell culture and in vivo with PDX models. Neither of these systems take into consideration the potential effects of a functional immune system on drug response, with respect to both tumor killing and tolerance of the host. Further studies using genetically engineered mouse models of PDAC will be essential to judge the clinical suitability of this therapeutic strategy. On this point, the use of BET inhibitors in the clinical setting is restricted because of the observed dose-limiting toxicities (*94*). However, we saw synergy across a range of JQ1 doses (Fig. 1G), suggesting that this combination may allow for lower dosing of BET inhibitors. It will be interesting to address the selective role of the bromodomain 1 (BD1) and bromodomain 2 (BD2) domains of BRD4 here further inaugurating potential strategies with lower toxicity and thus clinical applicability. Last, while we demonstrated that the SN38 + JQ1–induced DNA damage was depended on transcription using flavopiridol (Fig. 5), we were unable to specifically inhibit readthrough transcription without also blocking transcription in general. Therefore, we cannot completely exclude the possibility that some unknown transcription-dependent feature of SN38 + JQ1 treatment underlies the DNA damage response. As the readthrough transcription phenomenon continues to be elucidated by us and others, we hope to acquire the tools to further investigate the mechanism of action of combined BRD4 and TOP1 inhibition.

## MATERIALS AND METHODS

### Study design

The objective of this study was to test coinhibition of TOP1 and BRD4, which we previously demonstrated to be synergistic in vitro (*14*), in PDX models of pancreatic carcinoma to determine whether this treatment strategy could be effective in vivo. We hypothesized that targeting transcription through two independent arms would selectively kill tumor cells that are oncogenically addicted to transcription, while leaving normal cells unharmed. We also predicted that by targeting a fundamental feature of cancer proliferation (as opposed to a specific driver of oncogenesis), we would avoid the emergence of drug resistance often associated with targeted therapy. For this, we used a selection of PDAC and panNEC PDX models either treated acutely (4, 12, and 24 hours) to assess response to treatment or repeatedly stopping drug administration once the tumor has regressed and recommencing upon tumor progression, to establish whether the tumors remain sensitive to treatment. All transplanted mice were randomized into treatment groups. Mouse experiment end points were defined by the associated ethics approval to safeguard the health of the mice. The Bo103 PDAC was adapted for tissue culture to enable investigation of the mechanism of action in vitro. No data were excluded from these studies.

### Statistical analysis

All statistical tests were performed using GraphPad Prism version 8 or 9 or statistical functions in R using the tests described for each experiment. *P < 0.05, **P < 0.01, and ***P < 0.001. Information about statistical tests is provided in the figure legends for the respective figures and relevant Materials and Methods subsections.

### PDX biobank

Establishment of the PDX mouse model was performed using surgically resected PDAC and panNEC tissues collected from patients at the Ruhr-University Bochum Comprehensive Cancer Center. Informed and written consent was obtained from all patients. The study was approved by the ethics committee of the Ruhr University Bochum (permission nos. 3534-09, 3841-10, and 16-5792). Patient tumor tissues were xenografted in both flanks of nude mice (NMRI-*Foxn1nu/Foxn1nu*, Janvier, St Berthevin Cedex, France), expanded, isolated, and reimplanted for at least three generations. Five- to 10-week-old mice were housed under specific pathogen-free conditions where light, temperature (+21°C), and relative humidity (50 to 60%) were controlled. Food and water were available ad libitum. All animal experiments were approved by the local authorities (81-02.04.2017.A423) and performed in accordance with the guidelines for Ethical Conduct in the Care and Use of Animals. PDAC PDX class assignment was defined on the basis of consensus clustering on median-centered data for the top 3000 most variable features after signal normalization. To this end, non-negative matrix factorization was used for discovery and validated by consensus clustering with the help of the consensus cluster plus algorithm for determining cluster count and membership by stability evidence in unsupervised analysis. The algorithm begins by subsampling a proportion of items and a proportion of features from a data matrix. Each subsample is then partitioned into up to *k* groups by a user-specified clustering algorithm: agglomerative hierarchical clustering, *k*-means, or a custom algorithm. This process is repeated for a specified number of repetitions. Pairwise consensus values, defined as “the proportion of clustering runs in which two items are clustered together” (*95*), are calculated and stored in a consensus matrix for each *k*. Then, for each *k*, a final agglomerative hierarchical consensus clustering using distance of 1 − consensus values is completed and pruned to *k* groups, which are called consensus clusters. The algorithm was benchmarked with the Collisson subtypes, and they applied to the PDX data.

### Treatment cohorts

To establish treatment cohorts, tumor pieces (1 to 2 mm) from early passage PDXs (≤F4 generation) were soaked in undiluted Matrigel (Becton Dickinson) for 15 to 30 min and subsequently implanted subcutaneously onto female mice (NMRI-*Foxn1nu/Foxn1nu*, Janvier, St. Berthevin Cedex, France) at two sites (scapular region) using as many as four pieces per site. Tumors were allowed to grow to a size of approximately 100 to 200 mm^3^, at which time, mice were randomized in the treatment and control groups with five to six mice in each group. Tumor volumes were estimated from 2D tumor measurements by caliper measurements twice a week using the following formula: Tumor volume (mm^3^) = (π/6)(*D*)(*d*^2^), where *d* is the minor tumor axis and *D* is the major tumor axis. Mice were treated daily with JQ1 (Hycultec; 50 mg/kg) by intraperitoneal injection and with irinotecan (Hycultec) intraperitoneally three times per week (Monday, Wednesday, and Friday) at 15 mg/kg, followed by 1 week of treatment pause for two consecutive cycles. OTX015 (Hycultec) was given at 50 mg/kg, i.p., for five consecutive days, followed by 2 days of treatment pause.

### Multiplexed immunofluorescence histological staining

Multiplexed immunofluorescence (mIF) was performed using the Opal multiplex system (PerkinElmer, MA) according to the manufacturer’s instructions and as previously described (*96*). Briefly, formalin-fixed, paraffin-embedded (FFPE) sections were deparaffinized and then fixed with 4% paraformaldehyde before antigen retrieval by heat-induced epitope retrieval using citrate buffer (pH 6) or tris/EDTA (pH 9). Each section was put through several sequential rounds of staining; each includes endogenous peroxidase blocking and nonspecific protein blocking, followed by primary antibody and corresponding secondary horseradish peroxidase–conjugated polymer (Zytomed Systems or PerkinElmer). Each horseradish peroxidase–conjugated polymer mediated the covalent binding of different fluorophores using tyramide signal amplification. This covalent reaction was followed by additional antigen retrieval in heated citrate buffer (pH 6) or tris/EDTA (pH 9) for 10 min to remove antibodies before the next round of staining. After all sequential staining reactions, sections were counterstained with 4′,6-diamidino-2-phenylindole (DAPI) (Vector Laboratories). The sequential multiplexed staining protocol used primary antibodies for cleaved caspase 3, γH2AX and pan-Cytokeratin (PanCK), and with secondary antibodies labeled with Opal 570, 480, and 520, respectively. Slides were scanned and digitalized by Zeiss AxioScanner Z.1 (Carl Zeiss AG) with 10× objective magnification. Quantification of individual and/or coexpressing markers in the mIF images was performed with HALO (Indica Labs).

### Cell culture

To establish the primary cell line, the corresponding Bo103 PDX tumor was harvested and subsequently dissociated by gentleMACS dissociator (Miltenyi Biotec). The human tumor dissociation kit (Miltenyi Biotec) and mouse cell depletion kit (both from Miltenyi Biotec) were used according to the manufacturer’s protocol. Fibroblasts were removed by differential trypsinization. The identity of Bo103 was confirmed by comparing short tandem repeat (STR) data between the Bo103 PDX founder tumor and the derived primary cell line. STR analyses were performed as previously described (*97**)* and analyzed on a CEQ8800 sequencer (Beckman Coulter). Bo103 cells were grown in a 1:1 mix of high-glucose (4.5 g/liter) Dulbecco’s modified Eagle’s medium (DMEM) (Thermo Fisher Scientific, 61965059) and DMEM/F12 (Thermo Fisher Scientific, 11320033) containing 5% fetal bovine serum (FBS) (Thermo Fisher Scientific, 10270106), amphotericin (1.6 μg/ml; Sigma-Aldrich, A2411), 10 μM Y-27632 (Selleckchem, S1049), ciprofloxacin (6.2 μg/ml; Sigma-Aldrich, 17850), choleratoxin (8.4 ng/ml; Sigma-Aldrich, C8052), insulin (500 ng/ml; Sigma-Aldrich, I9278), and 20 nM 1-thioglycerol (Sigma-Aldrich, M6145) in a 37°C incubator supplied with 5% CO_2_. All in vitro drug treatments use 500 nM SN38 and 1 μM JQ1, unless otherwise noted. To enrich cells in S phase, cells were blocked in S phase by addition of 200 mM hydroxyurea for 18 hours. hTERT-HPNE cells were cultured in high-glucose DMEM containing 5% FBS, 2 mM GlutaMAX (Thermo Fisher Scientific, 35050061), epidermal growth factor (10 ng/ml; Thermo Fisher Scientific, PHG0314), and puromycin (750 ng/ml; Thermo Fisher Scientific, A11138-03) in a 37°C incubator supplied with 5% CO_2_.

### Antibodies

Primary antibodies used in this study are anti-Top1 (Abcam, ab109374), anti-BRD4 (Thermo Fisher Scientific, A301985A), anti-H3K36me3 (Abcam, ab9050), anti-H3K27ac (Abcam, ab177178), anti-CSTF64 (Thermo Fisher Scientific, A301092A), anti–RNAPII–Ser2-P (Abcam, ab5095), anti–total RNAPII (Abcam, ab817), anti-γH2AX for in vitro IF (Sigma-Aldrich, 05-636), anti-γH2AX for mIF (Cell Signaling Technology, 9718S), anti-p27 (Abcam, ab32034), anti-BrdU (Becton Dickinson, 555627), anti–cleaved caspase 3 (Cell Signaling Technology, 9664 L), and anti-PanCK (Abcam, ab6401). Secondary antibodies are anti-mouse (Thermo Fisher Scientific, A11029) and anti-rabbit (Thermo Fisher Scientific, A32790) Alexa Fluor 488–conjugated antibodies.

### Viability assay

Bo103 or hTERT-HPNE cells were plated in 96-well plates at 4000 cells per well. After 48 hours, cells were treated with 0.01 to 30 μM SN38 and JQ1 in a checkerboard format in a final volume of 200 μl. After a further 48 hours, medium was replaced with 50 μl of room-temperature medium, and the plates were incubated at room temperature for 30 min. Then, 50 μl of CellTiter-Glo 2.0 (Promega, G9242) was added to each well, the plates were placed on an orbital shaker for 2 min to lyse cells, and then well luminescence was measured using a FLUOstar Omega Microplate Reader (BMG Labtech). The relative viability was calculated by normalizing signal to the viability of medium-only wells. The Bliss coefficient was determined as the difference between the measured effect of the drugs and the predicted effect if the drugs act independently. This predicted effect was calculated as *d*_1_ *+ d*_2_ − *d*_1_*d*_2_, where *d*_1_ and *d*_2_ are the effect of each drug alone at the relevant concentrations.

### Quantitative polymerase chain reaction

Total RNA was extracted from Bo103 cells, using the NucleoSpin RNA Kit (Macherey-Nagel) according to the manufacturer’s instructions. cDNA synthesis was performed with 1 μg of RNA in a two-stage reaction, first annealing random primers (Promega) at 65°C for 5 min and then ice for 1 min, followed by reverse transcription using SuperScript IV Reverse Transcriptase (Invitrogen, Thermo Fisher Scientific) at room temperature for 10 min, following incubation at 50°C for 30 min, and then inactivation at 80°C for 10 min. Readthrough transcription analysis was performed with primers sets [*SERP1*, TCAGGACCTAGGTTTACTGAAGA (forward) and TCTTCTCTGCCCTAGCCCAA (reverse); *SEC61B*, CCTCCAGTTCTGGGTGGTTC (forward) and GACAGACAAGCCAGCAGCTA (reverse); *BPNT2*, TATCACCACCCTGCCTTGTG (forward) and TTGGCATCCATGCTCCGATT (reverse)] targeting specific regions approximately 60-kb downstream of the TES of the target gene. qPCR reactions were carried out on a CFX96 Real-time system device (BioRad Laboratories) using the Fast SYBR Green Master Mix (Applied Biosystems, Thermo Fisher Scientific), according to the manufacturer’s instructions. All reactions were run in duplicates in three independent experiments. The ΔΔ*C*_t_ method was used for data analysis and fold changes in gene expression were normalized relative to the DMSO-treated sample.

### Chromatin immunoprecipitation with reference exogenous genome

H3K27ac, H3K36me3, RNAPII–Ser2-P, total RNAPII, BRD4, TOP1, and CSTF64 ChIP were performed on Bo103 cells as described previously (*14*) with minor modifications. Chromatin from mouse embryonic fibroblast (MEF) cells was used to spike-in the chromatin from human Bo103 cells to enable normalization across samples. Briefly, Bo103 cells were treated for 4 hours with stated drug concentrations. Bo103 and MEF cells were cross-linked with 1% formaldehyde (Thermo Fisher Scientific, 28906) for 5 min. Cross-linking was stopped by the addition of glycine (125 mM), and cells were washed twice with cold phosphate-buffered saline (PBS). After harvesting cells by scraping, the pellet was washed once with PBS plus 0.5% bovine serum albumin and resuspended in radioimmunoprecipitation assay (RIPA) buffer [10 mM tris-HCl (pH 8.0), 1 mM EDTA (pH 8.0), 1% Triton X-100, 0.1% Na-deoxycholate, 0.1% SDS, and 200 mM NaCl, with the addition of protease inhibitor cocktail] to a final concentration of 1 × 10^7^ cells/ml. Samples were sonicated with Bandelin probe and Covaris ME220 sonicators to produce chromatin fragments of 400 base pairs (bp) on average. After centrifugation, extracts were immunoprecipitated. Two micrograms of anti–RNAPII–Ser2-P, 3 μg of anti–total RNAPII, 2 μg of anti-TOP1, 3 μg of anti-BRD4, 5 μg of anti-CSTF64, 2 μg anti-H3K27ac, or 1 μg anti-H3K36me3 was mixed with 35 μl of Protein A/G magnetic beads (Pierce, 88803) and incubated at 4°C for 6 hours with controlled rotation. For ChIP-Rx, Bo103 chromatin from 3 × 10^6^ to 10 × 10^6^ Bo103 cells was mixed with MEF chromatin at a 5:1 ratio. The mixture was incubated with Protein A/G–antibody complexes with rotation overnight at 4°C. Samples were washed twice with RIPA buffer, twice with RIPA buffer containing 300 mM NaCl, twice with LiCl buffer [10 mM tris-HCl (pH 8.0), 1 mM EDTA (pH 8.0), 250 mM LiCl, 0.5% NP-40, and 0.5% Na-deoxycholate], and twice with TE [10 mM tris-HCl (pH 8.0) and 1 mM EDTA (pH 8.0)]. The beads were then resuspended in 125 μl of TE plus 0.25% SDS supplemented with 60 μg of proteinase K (500 μg/ml; New England Biolabs, P8107S) and incubated overnight at 65°C. DNA was either recovered from the elute by phenol:chloroform:isoamyl alcohol (25:24:1) (Sigma-Aldrich, P2069) extraction followed by ethanol (EtOH) (100%) precipitation in the presence of 20 μg of GlycoBlue (Thermo Fisher Scientific, AM9515) or using the QIAquick PCR Purification Kit (QIAGEN, 28106) and dissolved/eluted in tris-HCl (pH 8.5). All ChIP-Rx experiments were performed in biological duplicates.

### Covalent adduct detection coupled to ChIP-seq for TOP1

Following Kuzin *et al.* (*32*), about 1 × 10^7^ Bo103 cells were treated (in biological duplicates) with SN38 alone or in combination with JQ1 for 1 hour. During the past 30 min, MG132 (10 μM) was added to the cells. Cells were immediately lysed in 2 ml of M buffer [9.3 mM tris-HCl (pH 6.5), 18.6 mM EDTA, 5.59 M guanidine thiocyanate, 0.93% dithiothreitol, 0.93% sarcosyl, and 3.72% Triton X-100] and briefly sonicated with Bandelin probe sonicator at 20% amplitude for 3 cycles with 30-s ON and 30-s OFF. DNA covalent adducts were precipitated with 50% EtOH at −20°C and centrifuged at 14,000 rpm, and pellets were washed thrice in wash buffer [20 mM tris-HCl (pH 7.5), 50 mM NaCl, 1 mM EDTA, and 50% EtOH]. Pellets were dried for 5 min and resuspended in TE–0.1% SDS [10 mM tris-HCl (pH 8.0), 1 mM EDTA (pH 8.0), and 0.1% SDS]. After 30-min incubation by gentle agitation, samples were further sonicated with Covaris ME220 sonicator for 5 min at High Cell protocol in milliTUBE–1 ml with AFA Fiber to produce fragments of about 1 kb. For the immunoprecipitation, 2 μg of anti-TOP1 was mixed with 30 μl of Protein A/G magnetic beads (Pierce, 88803) and incubated at 4°C for 6 hours with rotation. Beads were washed once with ice-cold PBS, and DNA covalent adducts from 1 × 10^7^ cells were added to the Protein A/G–antibody complexes and incubated overnight at 4°C with rotation. Washing was performed as described for the ChIP protocol but only once with every buffer and always in presence of 0.1% SDS. The beads were then resuspended in 100 μl of TE plus 0.5% SDS supplemented with proteinase K (500 μg/ml) and incubated for 4 hours at 65°C. Samples were then purified by QIAquick PCR purification kit.

### Fluorescence-activated cell sorting

Bo103 cells were treated for 6 hours with 500 nM SN38, 1 μM JQ1, 2 μM flavopiridol, or 15 μM XL-413. For the final 2 hours, cells were treated with 20 μM EdU to label replicating cells. Cells were harvested by trypsinization, washed in PBS, fixed by dropwise addition of chilled 70% EtOH while vortexing, and stored at −20°C. Cells were washed twice in PBSTB (PBS, 0.1% Triton X-100, and 1% bovine serum albumin), and then the EdU was conjugated to Alexa Fluor 647 via click chemistry using the Click-iT EdU Flow Cytometry Assay Kit (Invitrogen). The samples were washed in PBSTB and incubated with 1:1000 dilution of primary antibody at room temperature for 30 min and then washed and incubated with secondary anti-mouse or anti-rabbit antibody conjugated to Alexa Fluor 488 in the dark at room temperature for 30 min. Last, cells were washed and incubated in PBSTB with 1:1000 FxCycle Violet and ribonuclease (10 μg/ml) for 15 min in the dark to stain for DNA content. Samples were assayed on the fluorescence-activated cell sorting (FACS) Canto II (Becton Dickinson). Cells were categorized into cell cycle stages by DNA content and EdU positivity. All experiments were repeated three to four times.

### Microscopy

Bo103 cells were plated in 96-well tissue culture plates at 8000 cells per well and grown for 48 hours. Cells were treated for 6 hours with 500 nM SN38, 1 μM JQ1, 2 μM flavopiridol, or 10 to 15 μM XL-413. Cells were pulsed 15 min before drug treatment with 20 μM EdU, which was washed out 1 hour after drug treatment, and wells were replaced with drug-containing medium. The medium was aspirated, and cells were fixed with 3% paraformaldehyde/PBS at room temperature for 5 min before washing twice with PBS. Cells were permeabilized both by incubation at −20°C in methanol for 2 min and, subsequently after washing twice in PBS, by incubation in PBS + 0.5% Triton X-100 at room temperature for 10 min. The EdU was conjugated to Alexa Fluor 647 using the Click-iT EdU Flow Cytometry Assay Kit (Invitrogen), before the cells were blocked in PBSTB for 1 hour at room temperature. The wells were sequentially incubated for 1 hour with 1:250 primary γH2AX antibody and 1:1000 secondary Alexa Fluor 488 antibody with DAPI (1 μg/ml), with three PBSTB washes following each incubation. The plate was stored in PBS and imaged on the ImageXpress Micro Cellular Imaging System (Molecular Devices) and analyzed using CellProfiler 4 (*98*). Cells in S phase were identified based on EdU positivity. The experiment was independently repeated three times, demonstrating the same degree of significance between samples as the representative plot.

### Repli-seq

This method was adapted from a published protocol (*52*). Bo103 cells were treated in duplicate with vehicle, SN38, JQ1, or both drugs and then with 100 μM BrdU, 4 and 2 hours before harvest, respectively. Cells were trypsinized, resuspended in 2.5 ml of cold FACS buffer (PBS + 1% FBS), fixed with dropwise addition of 7.5 ml of ice-cold 100% EtOH while vortexing, and stored at −20°C. After washing twice in FACS buffer, 3 × 10^6^ cells were stained for DNA content in 300 μl of FACS buffer + propidium iodide (50 μg/ml) + ribonuclease (250 μg/ml). A total of 40 × 10^3^ to 120 × 10^3^ cells were sorted from the early S and late S populations for each sample. Cells were lysed in SDS-PK buffer [50 mM tris (pH 8), 10 mM EDTA, 1 M NaCl, 0.5% SDS, and Proteinase K (200 μg/ml)] at 56°C for 2 hours, and then DNA was extracted using the Zymo Quick-DNA Microprep Kit and eluted in 130 μl of H_2_O. DNA was sonicated to fragments of 200 to 300 bp in microtubes with the Covaris ME220 sonicator using the following settings: peak incident power, 70 W; duty factor, 20%; cycles/burst, 1000; time, 130 s. Each sample was ligated with adapters using the NEBNext Ultra II DNA Library Kit, and the adapter hairpins were processed with the USER enzyme and purified with the DNA Clean and Concentrator Kit (Zymo Research, D4013). DNA was denatured to single-stranded DNA by incubation at 95°C for 5 min and then on ice for 2 min. Each sample was diluted in immunoprecipitation buffer [10 mM sodium phosphate (pH 7), 140 mM NaCl, and 0.05% Triton X-100] and incubated with 0.5 μg of anti-BrdU for 20 min at room temperature while rocking. Protein A/G magnetic beads were added and followed by incubation for a further 30 min, before washing the beads twice with cold immunoprecipitation buffer. The DNA was eluted by incubation in digestion buffer [50 mM tris (pH 8), 10 mM EDTA, and 0.5% SDS] with 50 μg of Proteinase K overnight in a 37°C air incubator and then by adding a further 25 μg of Proteinase K and incubating at 56°C, shaking for 1 hour. The eluted single-stranded DNA was purified with the DNA Clean and Concentrator Kit (Zymo Research). All samples were amplified and indexed using the NEBNext Multiplex Oligos for Illumina, and primer dimers were removed by AMPure XP enrichment (Beckman Coulter) and sequenced using the NextSeq 500/550 High Output Kit v2.5 (Illumina, 20024907). The sequencing run was single end with 75-bp reads.

### SLAM-seq

A modified version of SLAM-seq (*40*) was used to measure both nascent RNAs and steady-state RNAs, over the full transcript length. A total of 1.5 × 10^6^ Bo103 cells were treated with DMSO, 500 nM SN38, 1 μM JQ1, or SN38 + JQ1 for 4 hours and in the past 2 hours. 4-Thiouridine (S4U; 100 μM) was added to the medium in the dark (in triplicates). Cells were scraped stepwise in 2 × 0.5 ml of TRIzol (Invitrogen, 15596018), snap-frozen, and stored at −80°C. For RNA isolation, we followed the manufacturer’s instructions of SLAM-seq Kinetics Kit–Anabolic Kinetics Module (Lexogen, 061.24) except for the following modifications: Frozen samples were thawed for 5 min at 65°C and mixed with 200 μl of chloroform:isoamyl alcohol mix (24:1) before further incubation for 15 min at 65°C (strongly shaking and vortexing from time to time). RNA in the aqueous phase was separated by centrifugation, supplemented with reducing agent and again extracted with chloroform:isoamyl alcohol mix (24:1) before precipitation overnight at −20°C (in the presence of reducing agent). RNA was washed twice with 1 and 0.18 ml of 75% EtOH (plus reducing agent), respectively, and pelleted for 10 min at 7500*g*. Resuspension in H_2_O was facilitated by incubation for 15 min at 55°C and 10 min on ice and repeated pipetting. RNA was quantified with the Qubit RNA Nano Assay Kit (Thermo Fisher Scientific, Q33230). RNA (4.5 μg) was alkylated by addition of iodoacetamide following the manufacturer’s instructions. Qubit RNA Nano Assay Kit (Thermo Fisher Scientific, Q33230) was used to quantify the RNA, and RNA integrity was controlled using the RNA 6000 nano kit (Agilent, 5067-1511) on a Bioanalyzer 2100 (Agilent). Ribosomal RNAs (rRNAs) were depleted from 950 ng of alkylated RNA using the RiboCop (HMR) kit (as part of the CORALL Total RNA-seq library Prep Kit, Lexogen, 146.24) following the manufacturer’s instructions, except for increasing the volume for RNA elution to 16 μl. Removal of rRNAs was confirmed using the RNA 6000 Pico Kit (Agilent, 5067-1413) on a Bioanalyzer 2100 (Agilent). To prepare cDNA libraries spanning the whole transcript length, the CORALL Total RNA-seq library Prep Kit (Lexogen, 146.24) was used, which includes reverse transcription with displacement stop primers. Ten microliters of the rRNA-depleted RNA was used as input material, and the final library was amplified by 14 PCR cycles. DNA concentration and molarity were determined by Qubit dsDNA HS Assay Kit (Thermo Fisher Scientific, Q33230) and Bioanalyzer High Sensitivity DNA Kit (5067-4626) on a Bioanalyzer 2100 (Agilent), respectively. Libraries were pooled and sequenced using the NextSeq 500/550 High Output Kit v2.5 (Illumina, 20024907). The sequencing run was single end with 75-bp reads.

### RNA harvest for sequencing from PDXs

PDX samples were taken from storage at −80°C and broken apart in liquid nitrogen using a mortar and pestle. The tumor fragments were then weighed in as to use at least 20 mg of tumor tissue and homogenized in either TRIzol reagent using a rotor stator instrument or crushing in the mortar and pestle with buffer from the AllPrep DNA/RNA Mini Kit (QIAGEN, 80204). RNA was extracted either using the TRIzol (Invitrogen, 15596018) or AllPrep kits. Afterward, 500 ng of RNA per sample was processed using the RiboCop kit (Lexogen, 037) for rRNA removal.

### Library preparation and sequencing of ChIP-Rx-seq and TOP1 CAD-seq

DNA from ChIP was quantified with the Qubit dsDNA HS Assay Kit (Thermo Fisher Scientific, Q33230). Sequencing libraries were created according to the ThruPLEX DNA-seq kit protocol (Takara, R400676). Size selection was performed in the range of 200 to 700 bp with AMPure XP beads (Beckman Coulter, A63880) and confirmed using the Agilent High Sensitivity DNA Kit (Agilent, 5067-4626) on the Agilent 2100 Bioanalyzer. Libraries were pooled and sequenced using the NextSeq 500/550 High Output Kit v2.5 (Illumina, 20024906). The sequencing run was single end and dual index with 75-bp reads. For the total RNAPII and TOP1 ChIP-Rx-seq, the libraries were pooled and sequenced using the NextSeq 1000/2000 P2 Reagents (100 cycles) v3 kit (Illumina, 20046811). These sequencing runs were single end and dual index with 115-bp reads.

### Library preparation and sequencing of RNA-seq and SLAM-seq

RNA extracted from xenografts or cultured cells was quantified with the Qubit RNA nano Assay Kit (Thermo Fisher Scientific, Q33230). For the Bo99 treated with irinotecan, JQ1, and irinotecan + JQ1, library preparation and sequencing were performed by Novogene. For all other sequencing, libraries were created according to the CORALL kit protocol (Lexogen, 095). Library preparation success was confirmed using the Agilent High Sensitivity DNA Kit (Agilent, 5067-4626) on the Agilent 2100 Bioanalyzer. Libraries were pooled and sequenced using the NextSeq 500/550 High Output Kit v2.5 (Illumina, 20024906). The sequencing run was single end and dual index with 75-bp reads for the in vitro SLAM-seq from cultured cells, while it was paired end and dual index with 75-bp reads each for the one from xenografts.

### ChIP-Rx-seq and CAD-seq data analysis

For the ChIP-Rx-seq and CAD-seq experiments, the generated fastq files were quality controlled with FastQC and MultiQC (*99*), trimmed with cutadapt, aligned to the human hg38 (and mouse mm10 for ChIP-Rx-seq) reference genomes with bowtie2 (*100*), deduplicated, sorted, and indexed using SAMtools (*101*) and Picard (*102*). BigWig files for visualization were generated using deepTools (*103*) using -scaleFactor option for ChIP-Rx-seq spike-in normalization. BigWig files of replicas were merged using UCSC bigwigmerge and bedGraphToBigWig for visual representation. The profiles of short-reads average distribution near TSSs and along normalized gene bodies were generated by ngs.plot (*104*), custom R scripts, Bioconductor (*105*) packages, and ggplot2. Spike-in normalization of average profiles was performed by multiplying the total number of reads by a factor inversely proportional to mouse spike-in unique deduplicated reads, and an average of two normalized replicas was used for average profile visualization. Only the protein coding genes from Ensembl 76 (*106*) database were used for generating the profiles. The profiles were smoothed using a local polynomial regression algorithm. The top 10,000 expressed genes were determined on the basis of the SLAM-seq from in vitro Bo103 cells treated with DMSO. The RNAPII–Ser2-P/total RNAPII–normalized profiles were generated by dividing the metagene profiles generated through ngs.plot by each other. Box plots were generated in R. Peaks were called from the H3K27Ac and H3K36me ChIPs using MACS2 (*107*). Using the ROSE algorithm (*38*, *108*), enhancers were then predicted from the H3K27Ac peaks called under the untreated (DMSO) condition. The RNAPII pausing index classified genes according to RNAPII recruitment (*109*, *110*). The pausing index is calculated to be the ratio of RNAPII reads density at the TSS over the average read density in the gene body. Pausing index calculations were performed in Python with custom scripts based on modified PIC software (*111*) and in R. Pausing index was calculated for all isoforms of expressed genes. Then, isoforms with the highest signal at the TSS, longer than 1 kb, and average RNAPII counts of >0 at TSS and gene body were selected for analysis.

### RNA-seq and SLAM-seq data analysis

For the modified RNA-seq and SLAM-seq experiments, the fastq files were prepared the same way as for the ChIP-Rx-seq data, but the RNA-seq reads were aligned to hg38 or mm10 reference genome using the splice-aware aligner HISAT2 (*112*). For deduplication, UMI_tools (*113*) was used, which allows for differentiation between PCR duplicates and naturally occurring deduplication due to the unique molecular identifier (UMI) added through the library preparation by CORALL kit (Lexogen, 095). For the Bo99 data treated with single or combined drugs, deduplication, sorting, and indexing were performed using SAMtools (*101*) and Picard (*102*). Reads mapping to both the human and mouse genome were excluded by aligning to one genome first and then using the unmapped reads of that run for alignment with the other genome (human or mouse). Aligned reads were split into two files containing either exonic or nonexonic reads. This was done by first generating a .bed-file of the exon annotations from a .gtf file of *Homo sapiens* transcriptome assembly release 36 (respectively release 25 for *Mus musculus*) from GenCode (*114*) using the grep and gtf2bed from the BEDOPS toolkit (*115*) commands in the shell. Using this .bed-file, the names of exonic reads were filtered from the respective .bam file using bam2bed and bedops also from BEDOPS, as well as awk in the shell. Last, using Picard tools’ command FilterSamReads, the .bam file was filtered to either include or exclude the list of reads overlapping exons generating two separate .bam-files containing exonic or nonexonic reads. To determine general gene expression level, RNA reads were counted using the featureCounts command from the Subread package (*116*). Subsequently, the read counts from the untreated (DMSO) condition in vitro were used to estimate general gene expression levels by averaging the fragments per kilobase million (FPKM) of triplicates. Bigwig files for RNA-seq visualization were generated using the bamCoverage command from the deepTools suite (*103*) after .bam-files had been merged for the in vitro RNA-seq using SAMtools merge (*101*).

For differential expression analysis, the R package “Index” (*117*) that is based on the “edgeR” package was used to account for differential expression in both exonic and intronic reads. Exons and introns were counted separately using the filtered .bam files (exonic and nonexonic) instead of assuming the intronic read count based on the difference between reads assigned to the entire gene body and the exons. Significantly differentially expressed genes (adjusted *P* < 0.05 and log_2_ fold change > 1 or < −1) were used for downstream analysis. For visualization of dependence of log_2_ fold change on length, lowly expressed genes (in either exonic or nonexonic reads) were excluded before calculating the moving average of the log_2_ fold change with a window of 250 genes below and above after ordering the genes by length. To create heatmaps, the R package “pheatmap” was used. The filtered nonexonic read files were also used for visualization of read distribution along the gene body and downstream of the TES (nonexonic reads distribution or NERD plots) via ngs.plot. The ngs.plot output was replotted in R and loess smoothed using the plot-default function. A small number of genes were excluded from the metagene plot as they caused spikes due to local high accumulation of reads. By applying a linear regression along the gene body, the SLAM and NERD indexes were calculated, which allowed for determination of change of RNA coverage along the gene body and its comparison between treatment conditions. For the in vitro SLAM-seq data, the binning method was changed to “bin” as opposed to the default “spline” to minimize the TES spike artifact.

GSEA preranked analysis (GseaPreranked) was performed with software version 4.2.3, using default settings except for “Collapse dataset to gene symbols” set to “No collapse.” Before analysis, a list was calculated with each gene assigned a log_2_ *P* value and ranked assigning a score based on the false discovery rate (FDR). The ranked genes are annotated according to the gene sets from Gene Ontology (GO) Biological Process, selected from the available in “gene sets database.” Gene sets identified as a significant FDR of <0.25 with GSEA.

To detect readthrough and DoG transcripts, the algorithm ARTDeco (*118*) was used on the merged .bam-files. To generate a more reliable set of readthrough genes, the default parameters were changed by extending the minimum length of initially detected DoGs to 12 kb and the DoG window size to 2 kb. After DoG detection, a set of high-stringency DoG was filtered from the DoGs detected after SN38 + JQ1 treatment by counting the RNA-seq reads covering the detected DoG regions split into windows of 5 kb for 100-kb downstream of the DoG transcript generating gene’s TES via featureCounts. The DoG genes were then filtered for those with a high level of readthrough [counts per million (CPM) > 1.5] 30- to 45-kb downstream of the TES. From the DoGs detected after SN38 + JQ1 treatment in vitro, a list of non-DoG genes with similar length and expression under this treatment condition was generated. The Venn diagram showing the overlap between in vivo and in vitro detected DoGs was made using the “Vennerable” package in R.

For analysis of nascent reads via the SLAM-seq method in vitro, the established pipeline slamdunk (*119*) was used. Since the sample preparation deviated from the standard SLAM-seq method, for instance, in the library preparation, the pipeline was adapted accordingly. Deduplication via UMI_tools (*113*) was performed after alignment via slamdunk map to the hg38 reference genome. After deduplication, slamdunk filter was run with a reference of the entire length of human genes as opposed to just the 3′ untranslated regions, followed by single-nucleotide polymorphism detection via slamdunk snp. Nascent reads with T > C transitions were then separated from background reads using the alleyoop read-separator command from the slamdunk pipeline. Nascent reads determined via T > C conversions were then visualized using ngs.plot as described above.

### Repli-seq data analysis

For the Repli-seq data, the generated fastq files were treated the same way as for the ChIP-Rx-seq files. However, after alignment and deduplication, the established protocol for Repli-seq analysis (*52*) was followed to determine read coverage in early and late S phase and the RT resulting from the ratio between the two (E, L, and E/L respectively). For visualization of RT downstream of the TES or at early and late replicated regions of DoGs and non-DoGs, a custom-made Python script was developed, before plotting the ensuing data in R with the default plotting function. For determination of early and late replicated regions, the algorithm RepliScan was used (*54*). The boundaries between those detected regions were then used to determine the distance from the TES of DoG genes detected in SN38 + JQ1 and the respective list of non-DoG genes in early regions. Chromosome 3 was excluded from this analysis as there was copy number variation in the 3q arm that interfered with RT calling. H3K36me peaks in late replicated regions were determined by taking the MACS2 called peaks and intersecting it with the determined late replicated regions via the intersect command from the BEDTools suite (*120*).

### Graphical design

Models for inhibitor function, readthrough, and replication stress induced by the treatment were created with BioRender.com

### GO enrichment analysis

GO enrichment analysis of biological processes was performed using the software PANTHER Classification System provided by GO web platform. For presentation, representative functional categories were selected on the basis of relevance from the hierarchical clustering tree obtained as an output.

## Supplementary Material

20231013-1
