# Supplementary material for: Coinhibition of topoisomerase 1 and BRD4-mediated pause release selectively kills pancreatic cancer via readthrough transcription
Source: Sci Adv. 2023 Oct 13;9(41):eadg5109. doi: 10.1126/sciadv.adg5109 (PMC10575591; doi:10.1126/sciadv.adg5109)
Supplement: Supplementary file 1 — Figs. S1 to S7 Legends for tables S1 to S6 [file sciadv.adg5109_sm.pdf]

Science Advances
AAAS

Supplementary Materials for

Coinhibition of topoisomerase 1 and BRD4-mediated pause release selectively kills pancreatic cancer via readthrough transcription

Donald P. Cameron *et al.*

Corresponding author: Laura Baranello, laura.baranello@ki.se; Stephan A. Hahn, stephan.hahn@rub.de

Sci. Adv. **9**, eadg5109 (2023)
DOI: 10.1126/sciadv.adg5109

The PDF file includes:

Figs. S1 to S7
Legends for tables S1 to S6

Other Supplementary Material for this manuscript includes the following:

Tables S1 to S6

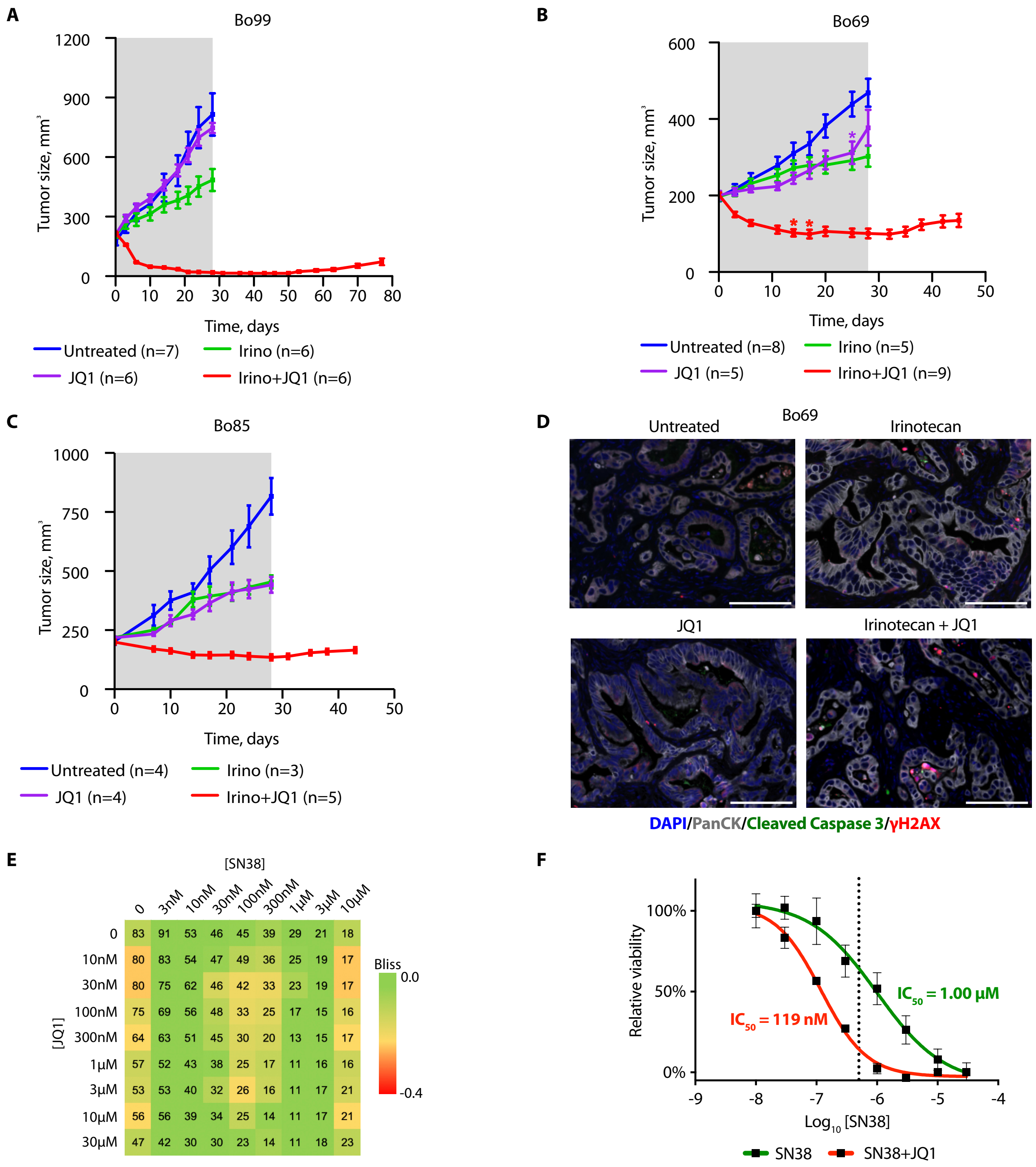

Fig. S1. Treatment with TOP1 and BRD4 inhibitors synergistically kills pancreatic tumor cells both *in vivo* and *in vitro*. (**A-C**) Primary responses observed within the 28 days treatment interval (grey shades area) for the panNEC model (A) Bo99, and the PDAC models (B) Bo69 and (C) Bo85 treated with Irinotecan (15 mg/kg, three times weekly, every second week) and JQ1 (50 mg/kg, daily) by i.p. injection, alone or in combination in comparison to untreated controls (cohort numbers shown). Growth curves are derived from mean values ± SEM (error bars). Each asterisk represents a mouse that was taken out of the treatment cohort (indicated by color) at the indicated time point because of health issues of the animal. (**D**) Representative immunohistochemistry images of Bo69 PDX tumor sections treated for 5 days with Irinotecan and/or JQ1 and stained for DNA (DAPI, blue), PanCK (grey), cleaved caspase 3 (green) and H2AX (red). Scale bar represents 100 m. (**E**) Checkerboard assay of cultured hTERT-HPNE cells treated with increasing concentrations of SN38 and JQ1 in combination as indicated. The percentage of confluency after treatment is denoted by the numbers in the squares. Synergy was determined using the delta Bliss model of additivity with lower, more negative values showing stronger synergy, (visualized by red/green color coding). Representative checkerboard of n=2. (**F**) Dose response curves showing cell viability 48 hr after treatment with 10 nM-30 M SN38 +/- 1 M JQ1 (n=3). IC_{50} values with 95% confidence interval – SN38: 1.00 M (0.53-1.89 M); SN38+JQ: 119 nM (78-182 nM).

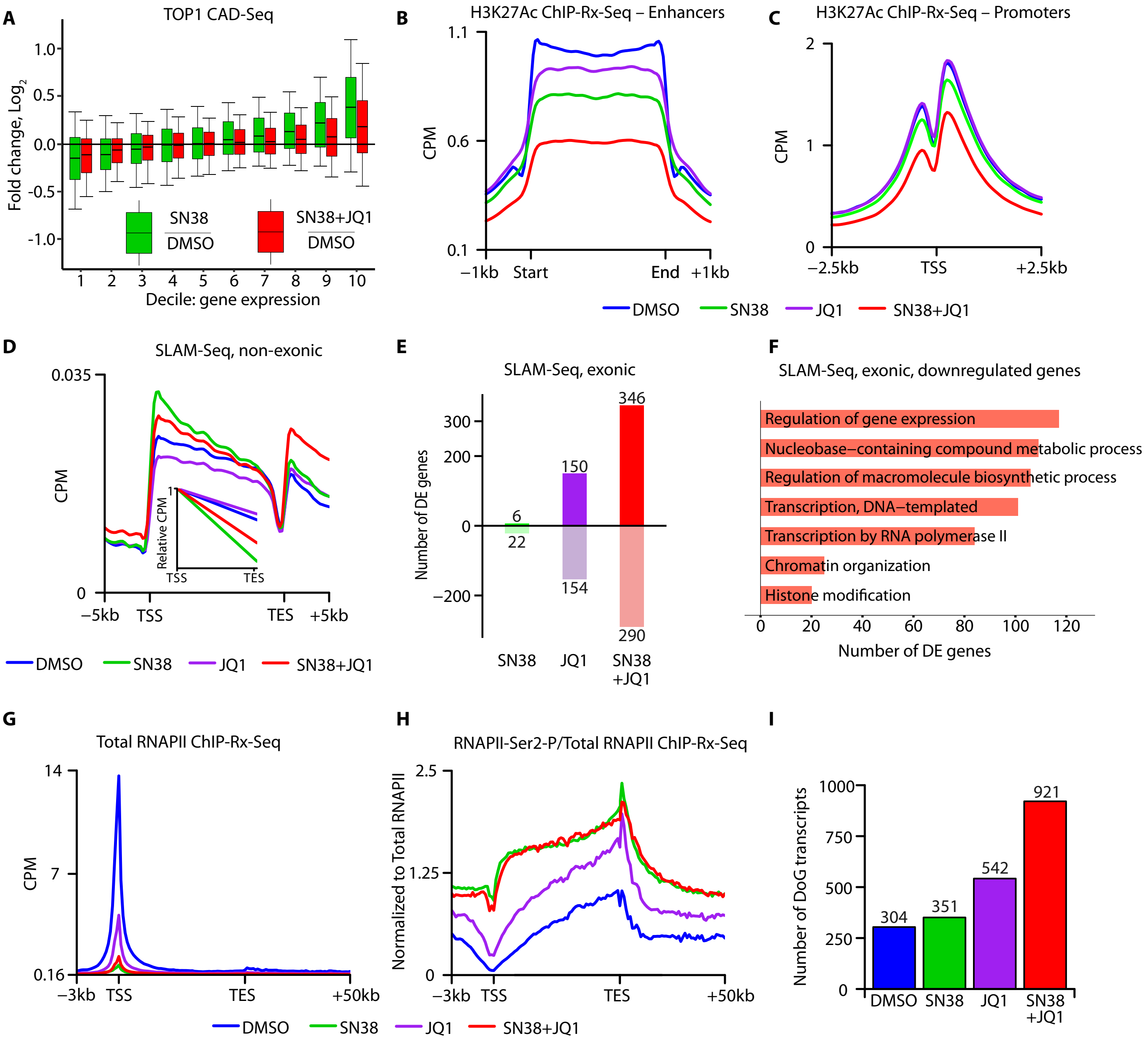

Fig. S2. Combination treatment SN38+JQ1 synergistically inhibits transcription. (**A**) Log_2 fold change of RPKM values between SN38/DMSO and SN38+JQ1/DMSO (derived from Fig. 2A TOP1 CAD-Seq) for each protein-coding gene binned in deciles by gene expression levels, whiskers extend to 10-90 %. (**B**) H3K27Ac occupancy at enhancers (+/- 1 kb) predicted from H3K27Ac peak calling (38, 108) in Bo103 cells treated with DMSO, SN38, JQ1, or SN38+JQ1 for 4 hr. Average of biological duplicates. (**C**) H3K27Ac occupancy at the TSS of the 10,000 most expressed genes (+/- 2.5 kb) in Bo103 cells after 4 hr treatment. Average of biological duplicates. (**D**) Non-exonic SLAM-Seq reads from Bo103 cells plotted between the TSS and TES of protein-coding genes after 4 hr treatment. Inset shows the gradient of the linear regression between TSS and TES (NERD index). Average of biological triplicates. (**E**) Number of significantly differentially expressed genes based on exonic reads of SLAM-Seq in Bo103 cells treated with SN38, JQ1, or SN38+JQ1 vs. DMSO-treated cells. (**F**) Gene ontology analysis of significantly downregulated genes (Log_2 fold change < -1, adjusted p-value < 0.05) from exonic reads of SLAM-Seq of Bo103 cells treated with SN38+JQ1 vs. DMSO-treated cells. (**G**) Total RNAPII occupancy at the 10,000 most expressed genes in Bo103 cells after 4 hr treatment. Average of biological duplicates. (**H**) Ratio of RNAPII-Ser2-P (Fig. 2G) to Total RNAPII (fig. S2G) at the 10,000 most expressed genes in Bo103 cells after 4 hr treatment. (**I**) Number of detected genes producing DoG transcripts after treating Bo103 cells with DMSO, SN38, JQ1, or SN38+JQ1.

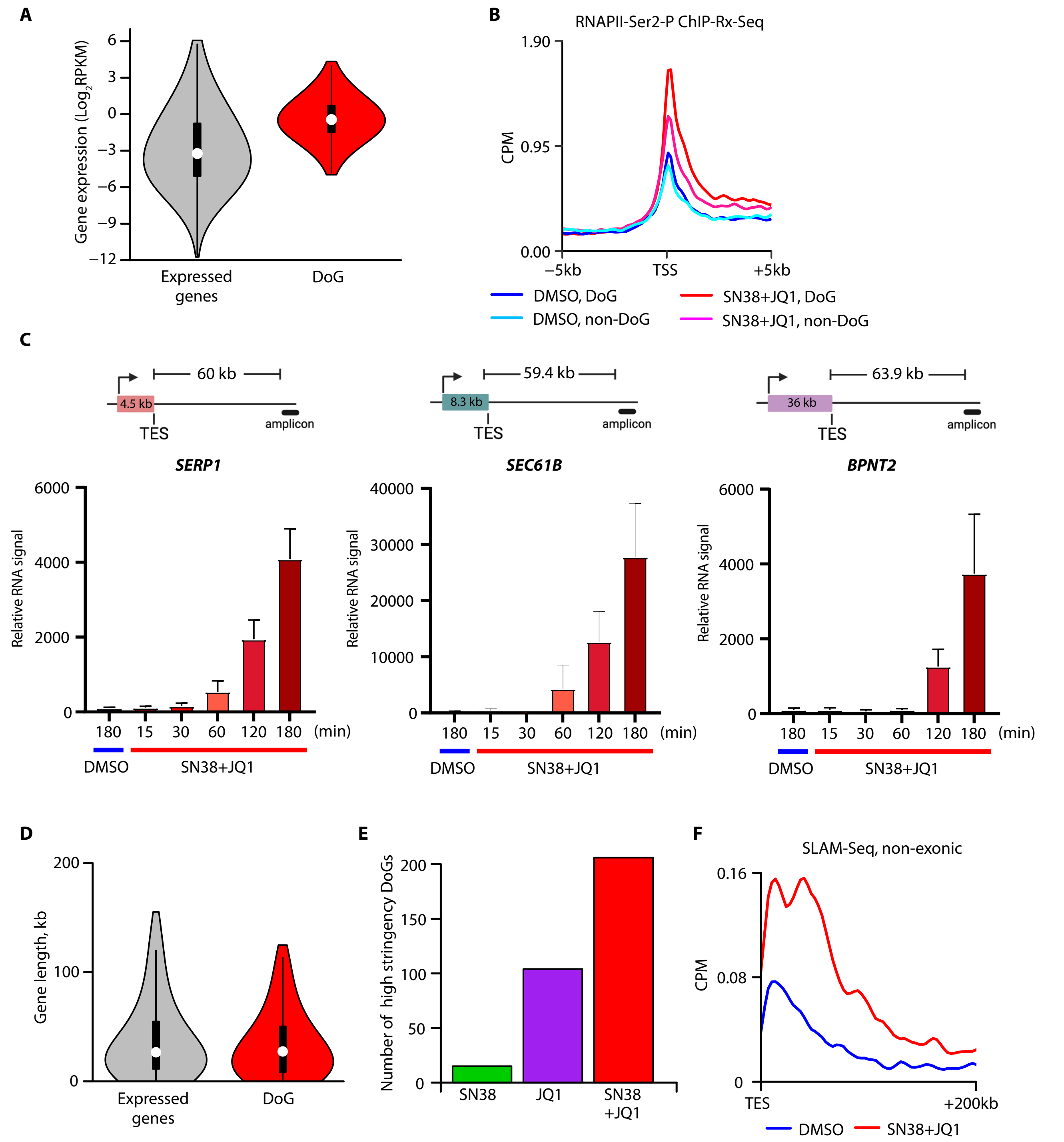

Fig. S3. Readthrough transcription is associated with defects in RNAPII pausing. (**A**) Violin plot of gene expression level of all expressed genes compared to DoG producing genes; outliers are excluded. (**B**) RNAPII-Ser2-P occupancy around TSS of DoG and non-DoG genes in Bo103 cells. Average of biological duplicates. (**C**) Top. Schematic showing distance between gene TES and qPCR amplicon. Bottom. Readthrough transcription downstream of DoG genes *SERP1*, *SEC61B* and *BPNT2* after 15 min up to 180 min of SN38+JQ1 exposure (n=3, relative to DMSO control, error bars represent standard deviation). (**D**) Violin plot of gene length of the top 10,000 expressed genes compared to DoG producing genes; outliers are excluded. (**E**) Number of DoG transcripts producing genes of Bo103 cells after filtering for high coverage above untreated levels 30-45 kb downstream of the TES. (**F**) Non-exonic SLAM-Seq reads from Bo103 cells plotted in the region 200 kb downstream of the TES of the high-stringency DoG genes in untreated (DMSO) or treated (SN38+JQ1) conditions. Average of biological triplicates.

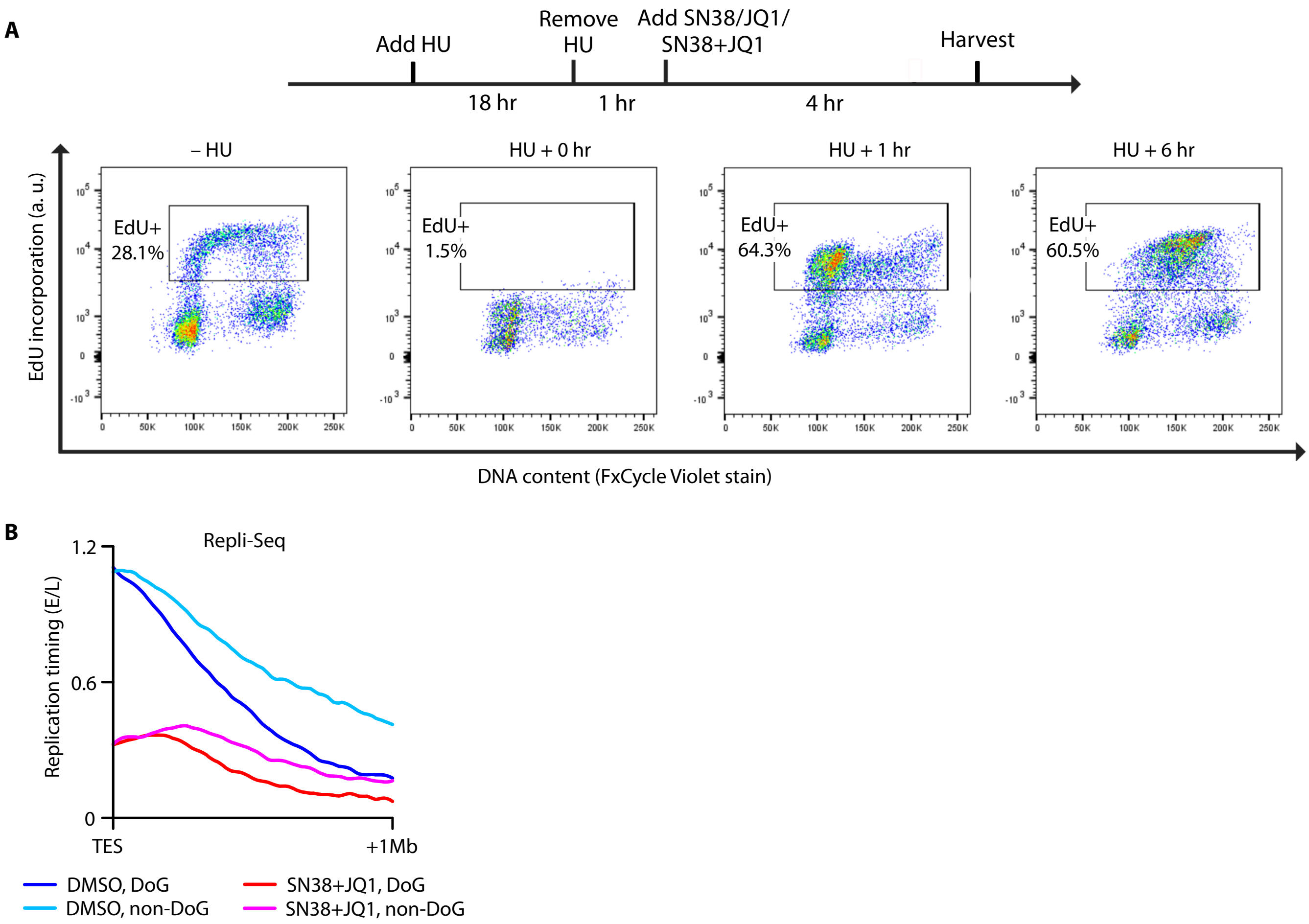

Fig. S4. SN38+JQ1-induced readthrough transcription extends from early to late replicated regions. (**A**) Scheme of S-phase synchronization by Hydroxyurea (HU) for Fig. 4A (top) confirmed by flow cytometry (bottom). Representative graphs of multiple time points showing the DNA content vs. EdU signal intensity, demonstrating that the majority of cells remain in S-phase up to 6 hr after HU removal. a.u. = arbitrary units. (**B**) Replication timing determined from Repli-Seq in Bo103 cells plotted 1 Mega base pairs (Mb) downstream of the TES of DoGs and non-DoGs in untreated (DMSO) or SN38+JQ1-treated conditions. The greater slope gradient of dark blue (DoG DMSO) vs. light blue (non-DoG DMSO) suggests that regions downstream of DoG genes, on average, transition from early to late replication timing sooner than non-DoG genes. Average of biological duplicates.

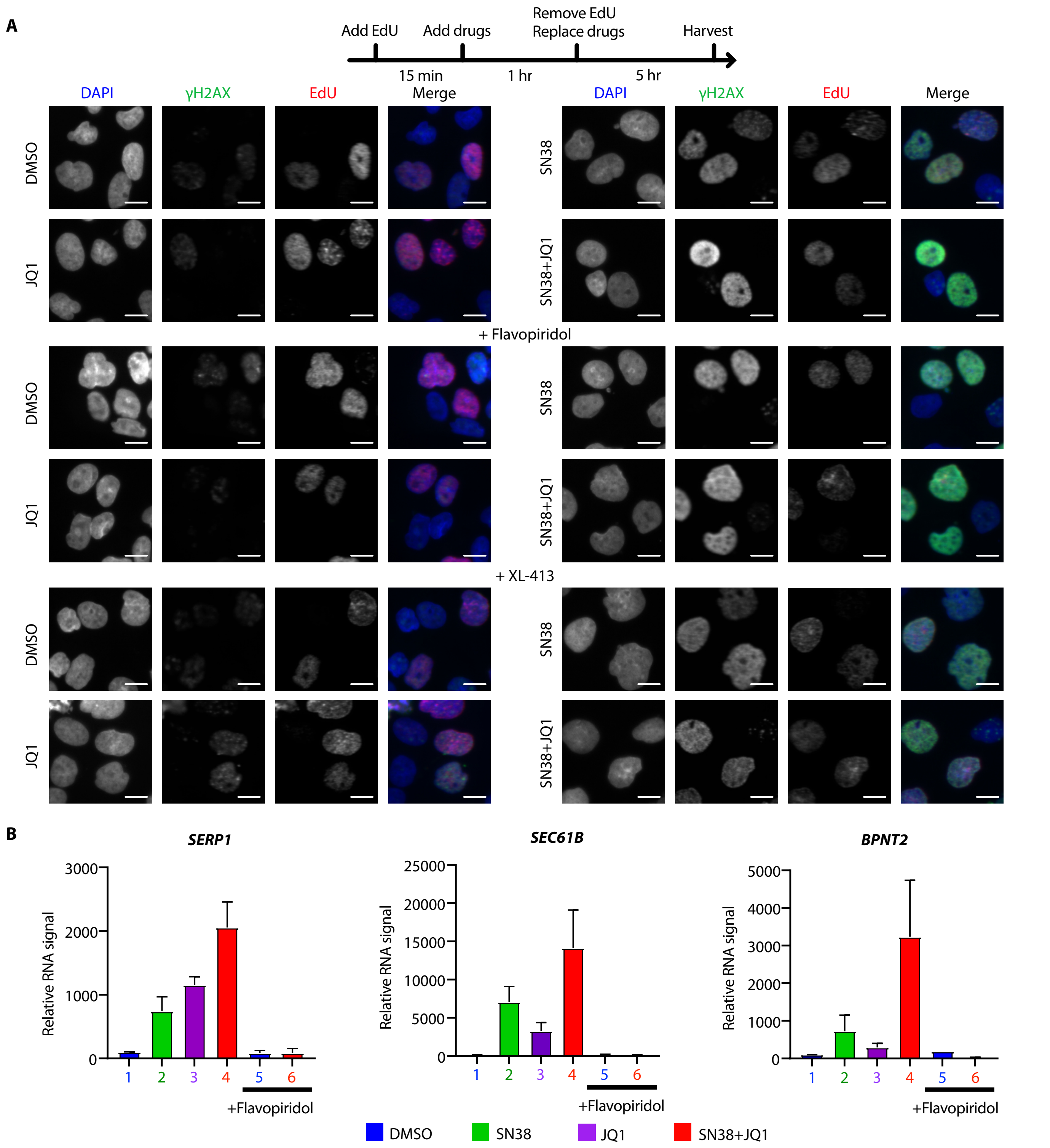

Fig. S5. SN38+JQ1-induced replication stress is dependent on ongoing transcription and replication. (**A**) Top. Schematic of treatment. Bottom. Representative images of Bo103 cells from Fig. 5A upon DMSO, SN38, JQ1, or SN38+JQ1 treatment +/- 2 M flavopiridol or 15 M XL-413 stained for DAPI (blue), H2AX (green) and EdU (red), shown individually and merged. Scale bar represents 10 m. (**B**) Readthrough transcription detected at the selected DoG regions downstream of the genes *SERP1*, *SEC61B* and *BPNT2* upon DMSO, SN38, JQ1, or SN38+JQ1 treatment +/- 2 M flavopiridol (n=3, relative to DMSO control, error bars represent standard deviation).

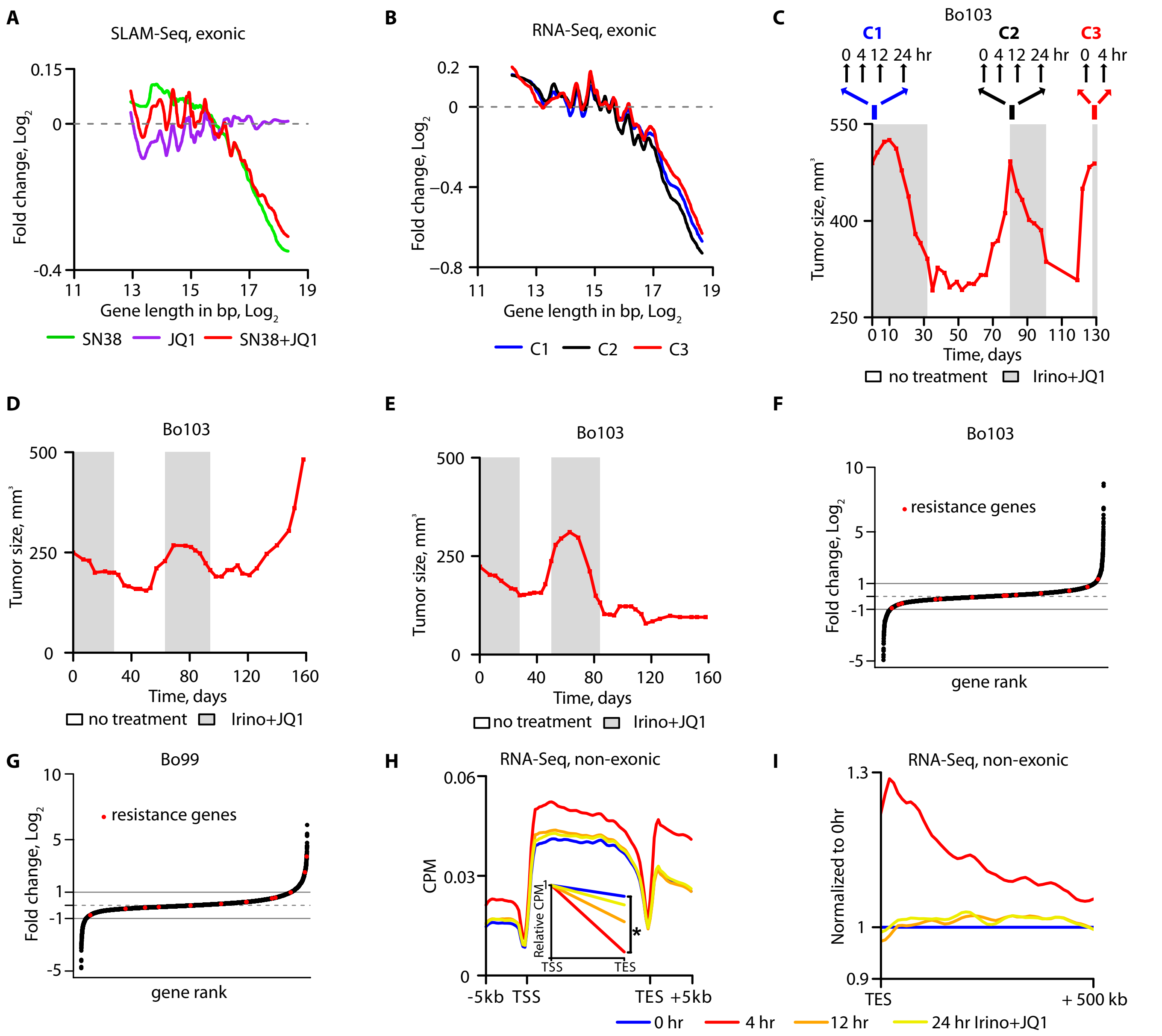

Fig. S6. Treatment of patient-derived xenografts (PDX) with Irinotecan+JQ1 triggers readthrough transcription without causing resistance over time. (**A**) Moving average of fold change (Log_2) from total exonic SLAM-Seq reads of treated (SN38, JQ1, or SN38+JQ1) vs. untreated Bo103 cells. Fold change is plotted against the gene length (Log_2). Average of biological triplicates. (**B**) Moving average of fold change (Log_2) of exonic RNA-Seq reads from Bo103 PDX treated with Irinotecan+JQ1 vs. untreated. Fold change is plotted against the gene length (Log_2). Drug cycles 1, 2 and 3 = C1, C2 and C3. Average of biological triplicates. (**C**) Top. Dosing and harvesting schedule. Bottom. Representative growth curve of an individual Bo103 PDX tumor, subjected to 3 cycles of treatment with Irinotecan+JQ1. (**D-E**), Representative growth curves of additional individual Bo103 PDX tumors, subjected to 2 cycles of treatment with Irinotecan+JQ1. (**F-G**), Ranked list of genes sorted and plotted by Log_2 fold change between C3 0 hr and C1 0 hr timepoints for (F) Bo103 and (G) Bo99 PDX tumors. Genes associated with resistance to Irinotecan or JQ1 treatment (Table S6) highlighted in red. (**H**) Non-exonic RNA-Seq reads from Bo103 PDX plotted between TSS and TES of protein-coding genes. Inset shows the slope of the curves between TSS and TES. Average of biological triplicates. *: $p<0.05$, Student's t-test. (**I**) Non-exonic RNA-Seq reads of Bo103 PDX plotted in the region 500 kb downstream of the TES of protein-coding genes. Data are expressed as counts per million (CPM) and normalized to the corresponding CPM values at 0 hr.

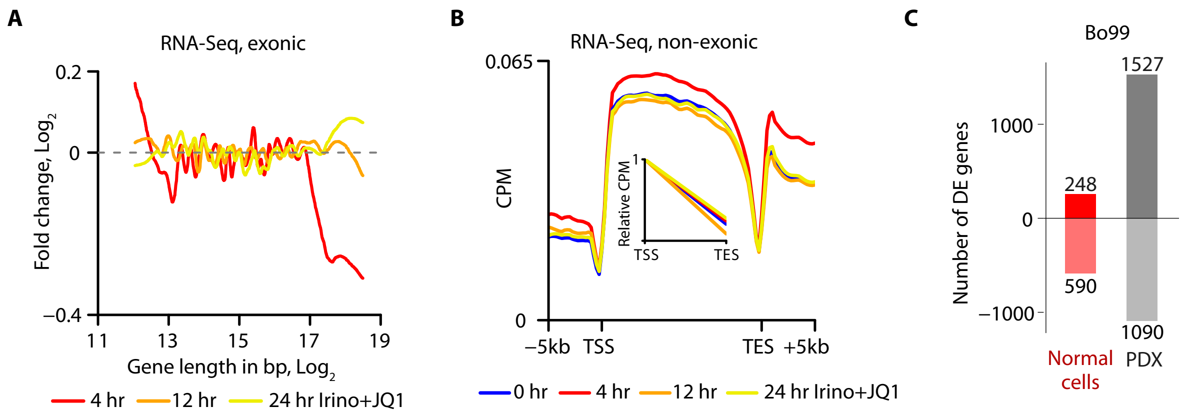

Fig. S7. Treatment with Irinotecan+JQ1 induces a stronger transcriptional response in Bo99 PDX than in normal mouse cells. (**A**) Moving average of fold change (Log_2) of exonic RNA-Seq reads from normal mouse cells derived from the Bo103 PDX in untreated vs. treated conditions (Irinotecan+JQ1 for 4, 12 and 24 hr). Fold change is plotted against the gene length (Log_2). Average of 3-4 tumors per condition. (**B**) Non-exonic RNA-Seq reads from normal mouse cells derived from the Bo103 PDX treated with Irinotecan+JQ1 for different times as indicated. Data are plotted between TSS and TES of protein-coding genes. Inset shows the slope of the curves between TSS and TES (NERD index). Average of 3-4 tumors per condition. (**C**) Bar plot representing the number of statistically significant (adjusted p-value < 0.05) differential expressed up- and down-regulated genes in Bo99 PDX and normal mouse cells upon treatment with Irinotecan+JQ1 for 4 hr.

Supplementary Table Legends (provided as Excel files)

Table S1.
PDAC subtype classification, KRAS mutation and MYC expression data for the PDAC and panNEC PDX models.

Table S2.
Differential expression analysis of exonic reads from *in vitro* Bo103 RNA-Seq with SN38, JQ1 and SN38+JQ1 treatments.

Table S3.
Differential expression analysis of exonic reads from *in vivo* Bo99 RNA-Seq with Irinotecan, JQ1 and Irinotecan+JQ1 treatments.

Table S4.
Differential expression analysis of human and mouse exonic reads from *in vivo* Bo99 RNA-Seq with Irinotecan+JQ1 timecourse treatments.

Table S5.
Differential expression analysis of human and mouse exonic reads from *in vivo* Bo103 RNA-Seq with Irinotecan+JQ1 timecourse treatments.

Table S6.
List of genes associated with resistance to either TOP1 or BRD4 inhibitions, highlighting differential expression (ΔLog_2 fold change) in Bo99 and Bo103 between C1 and C3 0 hr timepoints and mean expression levels (Log_2 FPKM). "Regulation" column indicates whether gene is reported to be upregulated or downregulated in resistant cells.
